# Chromatin assembly factor-1 preserves genome stability in *ctf4*Δ cells by promoting sister chromatid cohesion

**DOI:** 10.15698/cst2023.09.289

**Published:** 2023-08-14

**Authors:** Nagham Ghaddar, Pierre Luciano, Vincent Géli, Yves Corda

**Affiliations:** 1 Marseille Cancer Research Centre (CRCM), U1068 INSERM, UMR7258 CNRS, UM105 Aix Marseille Univ, Institut Paoli-Calmettes, Marseille, France. Ligue Nationale Contre le Cancer (Labeled Equip).

**Keywords:** CAF-1, Ctf4, replication, chromatin assembly, sister chromatid cohesion

## Abstract

Chromatin assembly and the establishment of sister chromatid cohesion are intimately connected to the progression of DNA replication forks. Here we examined the genetic interaction between the heterotrimeric chromatin assembly factor-1 (CAF-1), a central component of chromatin assembly during replication, and the core replisome component Ctf4. We find that CAF-1 deficient cells as well as cells affected in newly-synthesized H3-H4 histones deposition during DNA replication exhibit a severe negative growth with *ctf4*Δ mutant. We dissected the role of CAF-1 in the maintenance of genome stability in *ctf4*Δ yeast cells. In the absence of *CTF4*, CAF-1 is essential for viability in cells experiencing replication problems, in cells lacking functional S-phase checkpoint or functional spindle checkpoint, and in cells lacking DNA repair pathways involving homologous recombination. We present evidence that CAF-1 affects cohesin association to chromatin in a DNA-damage-dependent manner and is essential to maintain cohesion in the absence of *CTF4*. We also show that Eco1-catalyzed Smc3 acetylation is reduced in absence of CAF-1. Furthermore, we describe genetic interactions between CAF-1 and essential genes involved in cohesin loading, cohesin stabilization, and cohesin component indicating that CAF-1 is crucial for viability when sister chromatid cohesion is affected. Finally, our data indicate that the CAF-1-dependent pathway required for cohesion is functionally distinct from the Rtt101-Mms1-Mms22 pathway which functions in replicated chromatin assembly. Collectively, our results suggest that the deposition by CAF-1 of newly-synthesized H3-H4 histones during DNA replication creates a chromatin environment that favors sister chromatid cohesion and maintains genome integrity.

## INTRODUCTION

Nucleosome assembly during DNA replication is tightly coupled to ongoing DNA synthesis. Chromatin assembly factor-1 (CAF-1) is a conserved histone chaperone, essential for cell survival in multicellular organisms, that plays a key role in replication-dependent nucleosome assembly [1] [2] [3] [4] [5] and preserves genome stability [6] [7]. In budding yeast, CAF-1 consists of three subunits called Cac1, Cac2, and Cac3, which differ in their ability to bind H3-H4. Deletion of any CAF-1 subunits is viable but leads to multiple defects including replisome dysfunction and DNA damage sensitivity [3] [7] [8] [9] [10] [11] [12] [13]. The ability of CAF-1 to deposit H3-H4 onto replicating DNA depends on its physical interaction with PCNA, a processivity factor for DNA polymerases, which is localized at the sites of DNA synthesis during replication and repair [14] [15] [16] [17] [18].

Lysine 56 of histone H3 is transiently acetylated during S phase of the cell cycle and after DNA damage and is rapidly de-acetylated, by the action of the sirtuins Hst3 and Hst4, when cells enter the transition between G2 and M phases and after DNA repair [19] [20]. Histone H3 lysine 56 acetylation (H3K56ac) is mediated by the histone acetyl-transferase Rtt109 and the histone chaperone Asf1 [21] [22] [23] [24] leading to H3 and H4 ubiquitination by the Rtt101-Mms1-Mms22 E3 ligase complex [25]. H3-H4 ubiquitination promotes histones H3-H4 deposition at the fork proximity, coordinates nucleosome formation, and facilitates the stable progression of the replication fork [25] [26] [27]. Besides H3K56ac function in replication-coupled chromatin assembly, H3K56ac is also required for transcription, DNA repair-coupled chromatin assembly, inactivation of the DNA damage checkpoint, and meiosis [20] [28] [29] [30] [31] [32] [33] [34] [35]. H3K56ac together with H3K121,122 ubiquitylation mediated by Rtt101-Mms1-Mms22 promote sister chromatid cohesion (SCC), establishing a potential functional connection between histone deposition and cohesin activity [28] [36] [37].

Cohesion holds the two copies of the sister chromatids together from the moment of duplication to the onset of anaphase, subsequently, ensuring accurate chromosome segregation during mitosis [38] [39]. Sister chromatid cohesion is mediated at many points along the sister chromatids by the cohesin ring complex. In *Saccharomyces cerevisiae,* the cohesin ring complex consists of Smc1, Smc3, Scc1/Mcd1, and Scc3 subunits and is loaded onto the chromosomes by the Scc2-Scc4 deposition complex, in the G1/S phase, at broad nucleosome-free regions [40] [41] [42]. In addition, the cohesion ring complex preferentially accumulates at centromeres and between convergent transcribed genes [43] [44]. Scc2-Scc4 determines cohesins localization across the genome [41] [45] [46] [47] [48] [49] and is necessary for maintaining stable cohesion-DNA association during G1 [50]. Scc2-Scc4 directly interacts with the kinetochore protein Ctf19 at *CEN* loci. This interaction is dependent on Ctf19 phosphorylation by DDK, an important event for centromeric cohesion [51]. Subsequently, cohesins are converted to a tethering competent state through the action of the essential replication fork-associated acetyltransferase Eco1 that acetylates Smc3 at lysine 112 and lysine 113 [52] [53] [54] [55] leading to cohesion establishment during S phase [56] [57]. Recent studies revealed that Eco1-dependent Smc3 acetylation is promoted by transient DNA structures that form during Okazaki fragment maturation [58]. Cohesion generation can also be induced in G2/M when a DSB is present [45] [55] [59]. In anaphase, after the formation of the mitotic spindle, the Scc1 subunit is cleaved by separase, allowing sister chromatids segregation [60]. It is well established that PCNA interacts and recruits Eco1 to replication forks, where the levels of PCNA correlate with cohesion establishment [61]. Indeed, protein complexes such as Ctf18-RFC and Elg1-RFC, which influence PCNA loading and unloading, affect cohesion [62] [63] [64] [65]. Recently it has been shown that several factors associated with replication forks (Chl1, MCM, Bre1, and the ubiquitin ligase complex Rtt101-Mms1-Mms22) also recruit Eco1 and/or promote Eco1-dependent cohesion establishment during DNA replication [37] [39] [66] [67] [68]. In addition to Eco1 and the components of the cohesin ring complex, which are all required for viability, genetic analyses have identified an important number of replication proteins, functioning in S-phase, that mediate cohesion establishment. Two genetically distinct pathways, that involve multiple replication fork-associated proteins being non-essential for cell viability, contribute to cohesion establishment at the replication forks in yeast [69]. The first pathway is involved with Scc2 in the *de novo* loading of the nucleoplasmic cohesin pool at the site of DNA synthesis. It includes the S-phase checkpoint protein Mrc1, a core component of the replisome progressing complex required for normal replication fork progression [70] [71] [72], and the Ctf18-Ctf8-Dcc1 complex which forms an alternative replication factor C complex with Rfc2-Rfc5 (Ctf18-RFC) [73] [74]. The second pathway, independent of Scc2, is involved in conversion of the preloaded cohesin rings on the DNA template into a cohesive form. This pathway is composed of the replisome components Csm3, Tof1, Chl1, and Ctf4 [74]. Csm3 and Tof1 form the replication checkpoint complex with Mrc1 [75] [141]. Chl1 helicase controls replication fork progression [76], and physically engages with cohesin during cohesion establishment [66] [77]. Ctf4, which was identified in budding yeast as a chromosome transmission fidelity factor, is required for the maintenance of genome stability and SCC [56] [78] [79].

Ctf4 is a core component of the replisome progression complex [56] that forms a hub connecting replication forks to an important number of proteins [77] [80] [81]. During normal replication, Ctf4 recruits and stabilizes DNA polymerase-alpha at the replication forks and coordinates DNA unwinding and synthesis [82] [83] [84]. Furthermore, Ctf4 forms an axis with Mcm2 and Pol alpha to facilitate the transfer of parental H3-H4 to lagging strands [85]. Among various partners, Ctf4 interacts and recruits Chl1 to the replisome to coordinate replication fork progression and cohesion establishment [77]. Ctf4 also interacts with Mms22 to recruit the Rtt101-Mms1-Mms22 E3 ubiquitin ligase complex to the replisome during S-phase [86]. This interaction is important to maintain genome stability in presence of replicative stress through the H3K56ac-dependent CAF-1-independent pathway [82] [84] [87] [88] [89] [90] [91] [92] [93]. The Mms22/Ctf4 interaction also contributes to cohesion establishment by promoting Eco1 recruitment and stabilization at replication forks, and it has been proposed that Rtt101-Mms1-Mms22 E3 ubiquitin ligase acts through Ctf4 to coordinate replication coupled sister chromatid cohesion and H3K56ac-dependent nucleosome assembly [37].

We previously reported that during replicative stress, replisome function is modulated by H3K56ac and that Ctf4 is harmful upon DNA damage in the absence of the functional DNA repair/tolerance branch of the CAF-1-independent H3K56ac pathway [92]. In this study, we present pieces of evidence that H3K56ac and Rtt101-Mms1-Mms22 E3 ubiquitin ligase are not required for growth in the absence of Ctf4 contrary to CAF-1 and replication-coupled chromatin assembly. We report that the loss of CAF-1 function increases the cohesion defect observed in *ctf4*Δ cells, affects the Eco1-dependent Smc3 acetylation required for cohesion establishment, and provokes cell death in yeast cells affected in the major SCC establishment pathways. Taken together, our experiments are consistent with a model in which the nucleosome assembly function of CAF-1 is required to create an adequate structural environment required for sister chromatid cohesion establishment at DNA replication forks.

## RESULTS

### CAF-1 exhibits a synthetic genetic interaction with the replisome component Ctf4

**Figure 1:**
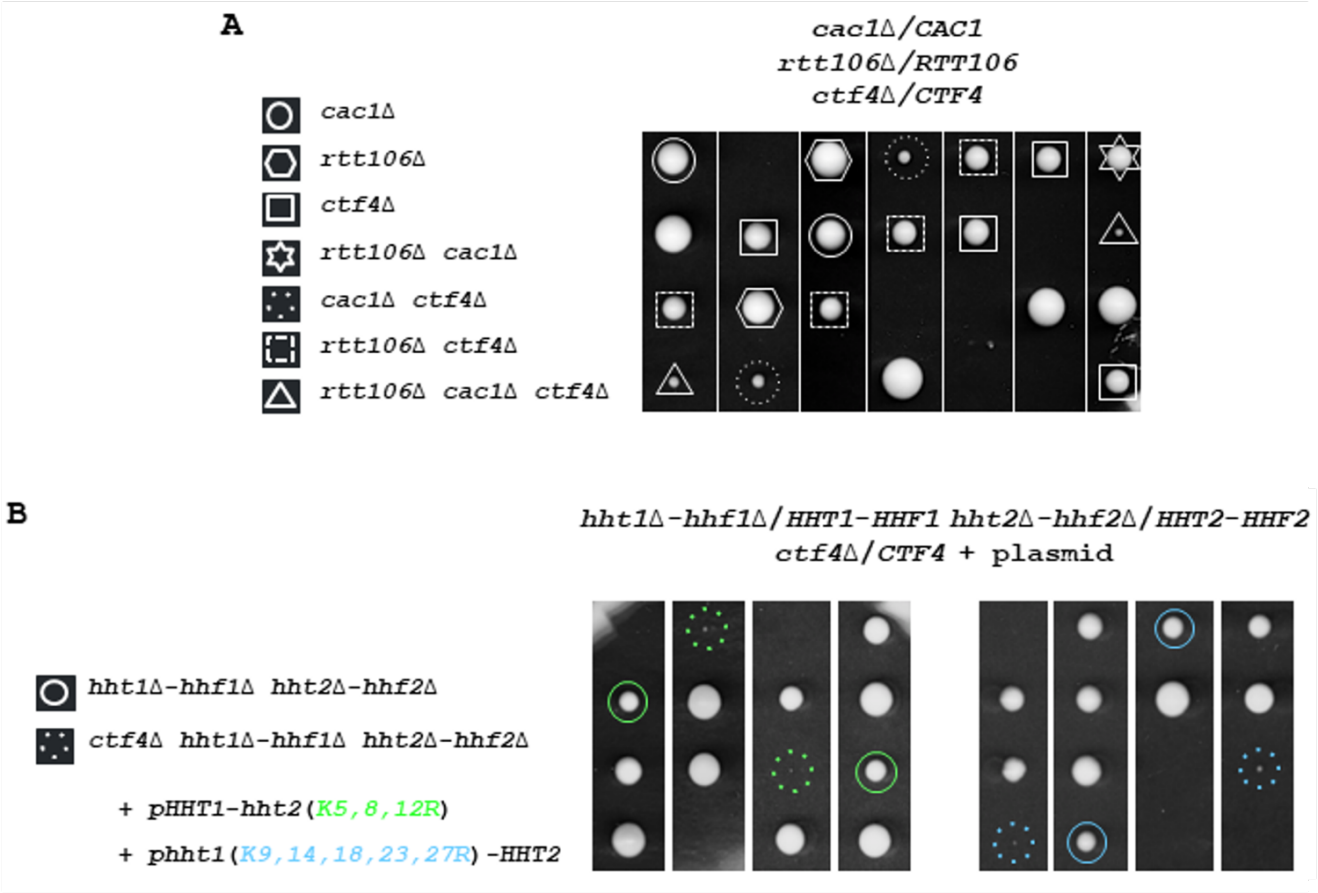
FIGURE 1: *CTF4* inactivation results in cell lethality in different genetic contexts affecting chromatin assembly. **(A)** Defective Cac1/Rtt106-dependent chromatin assembly affects growth in absence of *CTF4*. Tetrads from *rtt106*Δ/*RTT106 cac1*Δ/*CAC1 ctf4*Δ/*CTF4* diploid strain were dissected. In this and subsequent figures, the spores from a given tetrad are in vertical line in a YPD plate. Fifty tetrads were dissected. Five representative tetrads are shown after 3 days at 30°. **(B)** Mutations at histone lysine residues implicated in nucleosome assembly strongly affect growth of *ctf4*Δ cells. One hundred tetrads from diploids for *hht1*Δ-*hhf1*Δ/*HHT1-HHF1 hht2*Δ-*hhf2*Δ/*HHT2-HHF2 ctf4*Δ/*CTF4* expressing either *HHT2* and *hhf2*(*K5,8,12R*)*,* or *HHF1* and *hht1*(*K9,14,18,23,27R*) from a centromeric plasmid were dissected and analyzed for the presence of auxotrophic markers. The circle indicates spore expressing *H4K5,8,12R* (green), or *H3K9,14,18,23,27R* (blue) as the sole source of H4 or H3 histones, respectively. The dashed circle indicates *ctf4*Δ spore expressing *H4K5,8,12R* (green), or *H3K9,14,18,23,27R* (blue) as the sole source of H4 or H3 histones, respectively.

We have previously observed that the replication function of Ctf4 is strongly deleterious for yeast cells experiencing constitutive replicative damages in the absence of a functional H3K56ac-dependent pathway [92]. Having discovered that the effects observed are a direct consequence of the CAF-1-independent H3K56ac role in repairing/tolerating replicative DNA damage, we now investigate the consequences associated with *CTF4* inactivation in yeast cells defective in H3K56ac-dependent nucleosome assembly. H3K56ac facilitates replication-coupled chromatin assembly through a function that is dependent on CAF-1 and Rtt106 [25] [26] [27]. This pathway coordinates nucleosome assembly and stability of advancing replication forks but is not required for H3K56ac-mediated protection against replicative DNA-damaging agents by DNA repair/tolerance mechanisms [27] [94]. We carried out tetrad analysis after sporulating diploid heterozygous for *cac1*Δ *rtt106*Δ *ctf4*Δ. Dissection of meiotic tetrads shows that *cac1*Δ *rtt106*Δ *ctf4*Δ segregants grow at a very slow rate (**Figure 1A**). Interestingly, the growth of *cac1*Δ *ctf4*Δ double mutant was also considerably reduced and seemed slightly better than that of the triple mutant *cac1*Δ *rtt106*Δ *ctf4*Δ, suggesting that deletion of *RTT106* slightly exacerbates the growth defect of *cac1*Δ *ctf4*Δ cells. Finally, the growth difference between *rtt106*Δ *ctf4*Δ and *ctf4*Δ is much smaller than that between *cac1*Δ *ctf4*Δ and *ctf4*Δ, which means that *RTT106* was less required than *CAC1* in the absence of *CTF4* (**Figure 1A**). Taking into account that Cac1 and Rtt106 coordinate to deposit newly synthesized histone H3-H4 onto replicated DNA during S phase and DNA repair [95] [96], and based on the fact that Cac1 plays a most major role in this mechanism during DNA synthesis, these results suggest that the function of *CAC1* important for *ctf4*Δ cells growth is related to its chromatin assembly function. This function requires all CAF-1 subunits; Cac1, Cac2, and Cac3. Because CAF-1 subunits seem to have distinct functions in addition to their common nucleosome assembly function during DNA synthesis [97] [98] [99] [100] we analyzed the growth of *cac2*Δ *ctf4*Δ and *cac3*Δ *ctf4*Δ cells by monitoring the meiotic progeny of the diploid strains heterozygous for *CTF4* and *CAC2*, and *CTF4* and *CAC3*, deletions. We found that both *cac2*Δ and *cac3*Δ exhibited a strong negative interaction with *ctf4*Δ (Figure S1A). Moreover, we observed that *cac3*Δ *ctf4*Δ mutant was less affected in growth than *cac2*Δ *ctf4*Δ and *cac1*Δ *ctf4*Δ mutants (Figure S1A and Figure S2A, B, C). This observation could be explained by the fact that Cac3 depletion has a minor effect on nucleosome formation compared to *cac1*Δ and *cac2*Δ mutants [100]. Taken together these results strongly suggest that the nucleosome assembly function of CAF-1 during DNA synthesis is crucial in the absence of *CTF4*.

### Mutations affecting DNA-replication coupled nucleosome assembly exhibit a synthetic interaction with *ctf4*Δ

The HIR complex (formed by Hir1, Hir2, Hir3, and Hpc2) promotes replication-independent chromatin assembly [101]. This complex is important for normal growth and silencing in the absence of CAF-1, indicating functional overlap between HIR and CAF-1 complexes [102]. We found that *HIR1* was dispensable for the growth of *ctf4*Δ cells (Figures S1B and S2J), suggesting that the replication-independent chromatin assembly function is not important for *ctf4*Δ cells. We next conducted an extensive genetic analysis to assess the importance of genes encoding histone chaperones and histone acetyltransferases in *ctf4*Δ mutant cells (Figure S2). We first confirm that *RTT106* inactivation affected *ctf4*Δ cell growth less severely than *CAC1* inactivation (Figure S2E). Among many candidates tested we found that the Spt16 subunit of the heterodimeric FACT complex (Spt16-Pob3), which binds histone H3-H4 and Cac2 subunit of CAF-1, and functions in DNA replication-coupled nucleosome assembly [103], exhibited negative genetic interactions with *ctf4*Δ (Figure S2H). In addition, we show that *rfa1-A88P* mutation, which exhibits attenuated nucleosome assembly on nascent chromatin [104] was lethal with *ctf4*Δ (Figure S2K). Finally, we observed that the lysine acetyltransferase Gcn5, which regulates the interaction between H3-H4 and CAF-1 to promote the deposition of newly-synthesized histones [105] was also important for the normal growth of *ctf4*Δ cells (Figure S2M). We noticed that *asf1*Δ as well as *rtt109*Δ only slightly affected *ctf4*Δ cells (Figure S2D and L). H3 and H4 N-terminal tail acetylation serve as an important regulator of nucleosome assembly [26] [105] [106] [107]. We therefore, determine the importance of different H3-H4 histone modifications involved in nucleosome assembly, in *ctf4*Δ cells.

We next crossed a strain expressing *H3K9,14,18,23,27R* or *H4K5,8,12R* from a centromeric plasmid as the sole source of histone H3 or H4 with the *ctf4*Δ strain and analyzed the spores after diploids sporulation. We observed that in the absence of *CTF4*, the mutation at H3 or H4 lysine residues, implicated in nucleosome assembly, became deleterious (**Figure 1B**). We further analyzed the consequences of the absence of Nap1, which promotes H2A-H2B tetramer assembly in nucleosomes, in *ctf4*Δ cells. We found that *nap1*Δ did not negatively affect the growth of *ctf4*Δ cells (Figure S1C). Altogether, these genetic analyses indicate that defects in chromatin assembly during replication related to H3-H4 histones are deleterious in absence of *CTF4.* These results further support the notion that CAF-1 has a crucial role in a process linked to the Ctf4 function.

### Cac1 is important for genome integrity in absence of *CTF4*

**Figure 2:**
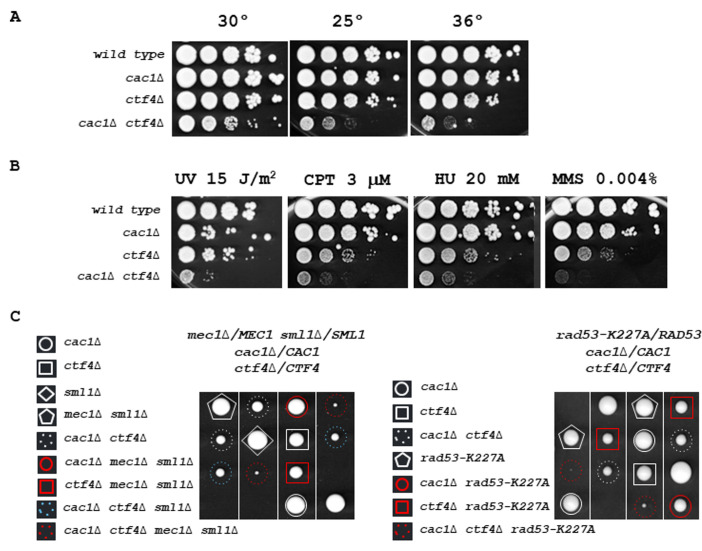
FIGURE 2: *CAC1* is important for growth in the absence of *CTF4*. **(A)** *cac1*Δ *ctf4*Δ growth is affected at various temperatures. Tenfold serial dilutions of wild-type, *cac1*Δ, *ctf4*Δ, and *cac1*Δ *ctf4*Δ cells were spotted onto YPD plates and incubated at 30° (left), 25° (middle), or 36° (right) for 3 days. **(B)** *cac1*Δ *ctf4*Δ growth is affected in presence of DNA damage. Tenfold serial dilutions of wild-type, *cac1*Δ, *ctf4*Δ, and *cac1*Δ *ctf4*Δ cells were assayed on normal growth media (YPD), after UV irradiation or not, and on media containing the indicated DNA-damaging agents, camptothecin (CPT), hydroxyurea (HU), and methyl-methanesulfonate (MMS). **(C)** The S-phase checkpoint is required for *cac1*Δ *ctf4*Δ mutant viability. The diploid strains *mec1*Δ*/MEC1 sml1*Δ*/SML1 cac1*Δ/*CAC1 ctf4*Δ/*CTF4* (left) and *rad53-K227A/RAD53 cac1*Δ/*CAC1 ctf4*Δ/*CTF4* (right) were sporulated and one hundred tetrads were dissected on YPD plates and incubated at 30° for 5 days. Four representative tetrads are shown for each dissection. *mec1*Δ *sml1*Δ and *rad53-K227A* mutations are lethal in *cac1*Δ *ctf4*Δ cells.

To eliminate the possibility that *cac1*Δ *ctf4*Δ growth defects originate from meiotic events, we deleted *CAC1* in the *ctf4*Δ strain background by gene targeting. We first confirmed the slow growth phenotype, observed for *cac1*Δ *ctf4*Δ, during the segregation analyses and observed that this phenotype is amplified at both 25°C and 35°C (**Figure 2A**). We next examined the sensitivity of *cac1*Δ *ctf4*Δ mutant to both ultraviolet (UV) light and chronic exposure to DNA-damaging agents. We found that the *cac1*Δ *ctf4*Δ double mutant was more sensitive to UV, camptothecin (CPT), hydroxyurea (HU), and methyl-methanesulfonate (MMS) compared to every single mutant (**Figure 2B**). Then, we assessed in *ctf4*Δ mutant, the importance of Cac1 in the absence of Rrm3 which facilitates the progression of replication forks through non-histone DNA-protein complexes [108] [109]. We found that Cac1 was crucial for *rrm3*Δ *ctf4*Δ cells (Figure S3). These results indicate that replication stress and replication-induced DNA damage are lethal for *cac1*Δ *ctf4*Δ mutant. We next evaluated if the absence of Cac1 in *ctf4*Δ cells cause a synthetic interaction with mutations affecting the S-phase checkpoint pathway. We found that the absence of Mec1 or Rad53 kinases, that activate both branches of the S-phase checkpoint pathway [110], strongly affect the viability of *cac1*Δ *ctf4*Δ cells (**Figure 2C**).

**Figure 3:**
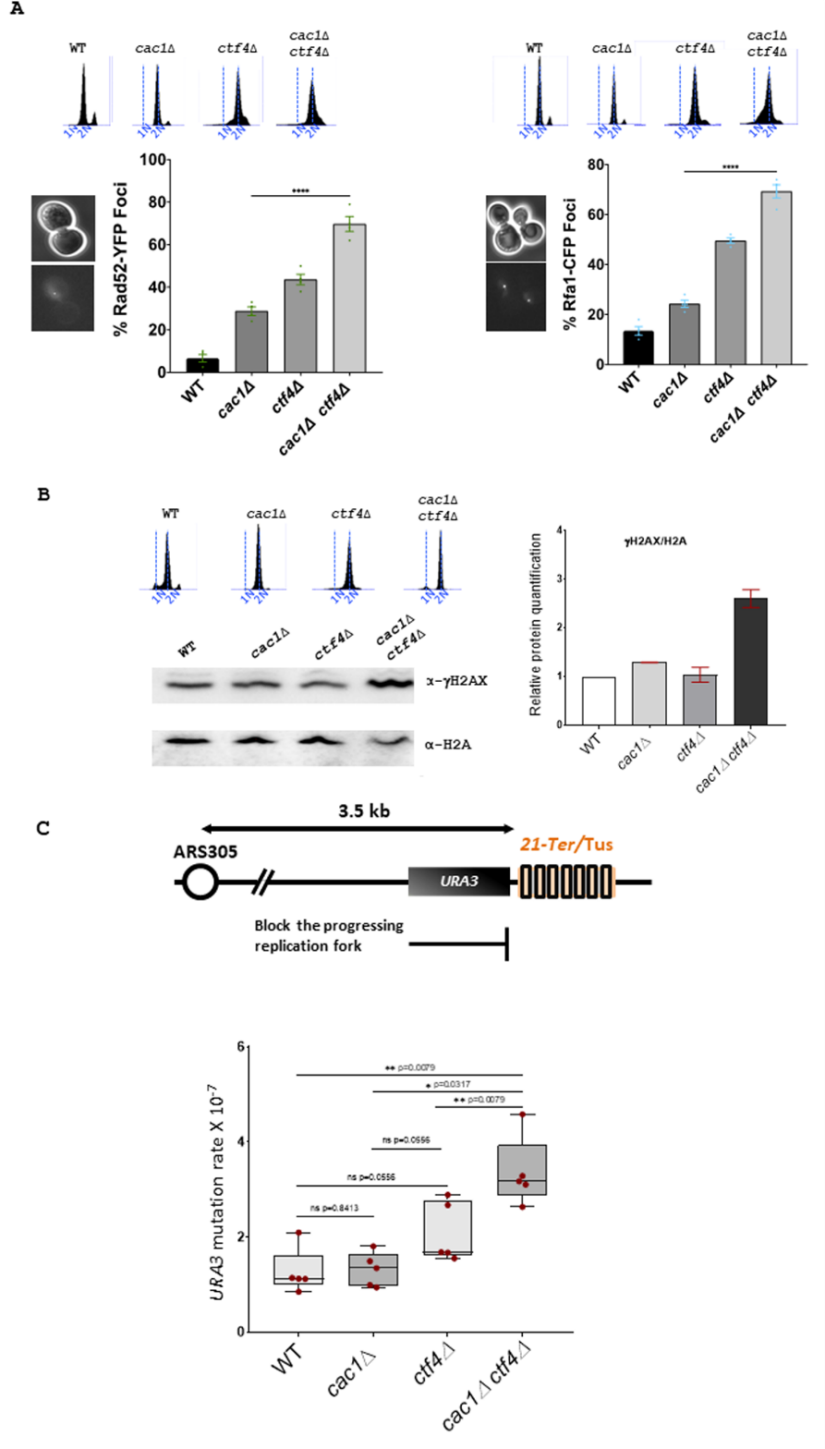
FIGURE 3: Genomic integrity is affected in *cac1Δ ctf4Δ* cells. **(A)** Left, Rad52 foci are increased in *cac1*Δ *ctf4*Δ cells. Wild-type, *cac1*Δ, *ctf4*Δ, and *cac1*Δ *ctf4*Δ cells encoding Rad52-YFP were analyzed with fluorescence microscopy. Right, Rfa1 foci are increased in *cac1*Δ *ctf4*Δ cells. Wild-type, *cac1*Δ, *ctf4*Δ, and *cac1*Δ *ctf4*Δ cells encoding Rfa1-CFP were analyzed with fluorescence microscopy. Numbers indicate the percentage of cells that contained Rad52-YFP (left) or Rfa1-CFP foci (right). DNA replication was monitored by FACS analysis of DNA content. At least 200 cells were analyzed for each strain from three independent experiments. Statistical significance was measured using the two-tailed Mann-Whitney test. **(B)** γH2AX is increased in the absence of Cac1 in *ctf4*Δ cells. Left, Western blot was used to detect phosphorylation of H2A serine 129 (γH2AX). Right, histogram shows for WT, *cac1*Δ, *ctf4*Δ, and *cac1*Δ *ctf4*Δ, γH2AX/H2A ratios calculated based on Western blots signal intensities. DNA replication was monitored by FACS analysis of DNA content. The experiment has been done in duplicate. **(C)** CAF-1 inactivation increases mutation rate in *ctf4*Δ cells at the Tus/*Ter* barrier. Top, Schematic representation of the unidirectional and site-specific Tus-*Ter* replication fork barrier. *Ter* sequence is integrated 3.5 kb downstream ARS305 on Chromosome III where Tus protein (colored rectangles) binds specifically to *Ter* sequence, causing replication fork pausing. Upstream to Tus/*Ter* replication fork barrier is the *URA3* reporter gene, which permits the positive selection for *ura3* mutations in presence of 5-FOA to measure mutation rate. Bottom, exponentially growing cells expressing Tus protein were plated for 3 days at 30° on YPGal plates and plated out on 5-FOA to select for *ura3* mutation. Box-and-whisker plots, representing the upper and lower quartile with the median, show the mutation rate in WT, *cac1*Δ, *ctf4*Δ, and *cac1*Δ *ctf4*Δ cells. Statistical analyses were done on n=5 independent experiments using two-tailed Mann-Whitney test; *p<0.05; ** p< 0.005; **** p<0.0001; ns, not significant.

To evaluate if homologous recombination (HR) was important for *cac1*Δ *ctf4*Δ mutant, we investigated whether *cac1*Δ *ctf4*Δ leads to an increase in spontaneous Rad52 foci, which reflect HR proteins recruitment into repair foci [111] [112]. We found that *cac1*Δ *ctf4*Δ cells exhibited a higher frequency of Rad52-YFP foci compared to *cac1*Δ and *ctf4*Δ single mutants (**Figure 3A**, left). We also found that *cac1*Δ *ctf4*Δ exhibited an abnormally high frequency of spontaneous Rfa1 foci compared to that in *cac1*Δ and *ctf4*Δ single mutants (**Figure 3A**, right). These results suggest that the absence of Cac1 spontaneously creates chromosome breaks or ssDNA gaps during replication in *ctf4*Δ cells and that these damages, are repaired by HR.

In *S. cerevisiae*, phosphorylation of histone H2A on serine 129 (γH2AX) is tightly associated to DNA damage, and it has been shown that DNA double-strand breaks levels can be obtained by measuring levels of γH2AX [113] [114]. To determine if the absence of Cac1 increased DNA damage in *ctf4*Δ cells, we analyzed by Western blot γH2AX levels, after a nocodazole-imposed mitotic arrest, (**Figure 3B**). In agreement with previously published results showing that *cac1*Δ mutant had only a weak requirement for replication or DNA-damage checkpoint proteins [99], we found a slight increase of H2A phosphorylation in absence of Cac1 compared to wild-type (WT) cells. In contrast, we observed a more consistent increase over WT cells in *cac1*Δ *ctf4*Δ mutant, indicating that the absence of Cac1 induces DNA damages in *ctf4*Δ cells.

Having found that Cac1 was required for the viability of *ctf4*Δ mutant in absence of Rrm3 helicase (Figure S3), which helps replication fork traverse protein-DNA complexes [108] and assists fork progression across *TERs* [109], we sought to evaluate whether loss of CAF-1 function affected genome stability in *ctf4*Δ cells. For this end, we used the natural *Escherichia coli* Tus/*Ter* barrier system known to induce an unidirectionally and site-specific replication fork stalling in *S. cerevisiae*. Interestingly, this system, which reflects the natural protein-bound DNA barriers arising in yeast cells, represents one of the most physiological replicative stresses that yeast cells could encounter [115] [116] [117]. The Tus/*Ter* barrier system, composed by a 21-bp DNA sequence (*Ter*) which is bound by the Tus terminator protein, is coupled with the genetic *URA3* gene reporter located immediately upstream the Tus/*Ter* barrier (**Figure 3C**, top), allowing us to quantify mutagenic outcomes as previously described [118] (Ghaddar *et al* 2023 Nat Commun, in press). In correlation with our previous data showing an increased Rad52 and Rfa1 foci formation and an increased level of γH2AX in *cac1*Δ *ctf4*Δ double mutant, we observed an increase in mutation rate in *cac1*Δ *ctf4*Δ cells compared to *cac1*Δ and *ctf4*Δ cells expressing Tus protein (**Figure 3C**, bottom).

Finally, we found that the elimination of Mad2 spindle checkpoint, which causes impairment of microtubule-kinetochore attachment and incomplete sister chromatid cohesion, negatively affects the growth of *cac1*Δ *ctf4*Δ cells (Figure S4). Overall, these results reflect the requirement of both DNA damage and spindle checkpoint for the viability of *cac1*Δ *ctf4*Δ cells and that HR is required for cell growth in the absence of both *CAC1* and *CTF4*. They show that loss of CAF-1 function causes DNA damages and leads to spontaneous mutation in *ctf4*Δ cells, revealing that CAF-1 prevents DNA damage formation and maintains genome stability.

### Dissecting the importance of Cac1 in functions mediated by Ctf4

Ctf4 performs different functions in DNA metabolism. To explore the significance of the negative genetic interaction between *CAC1* and *CTF4*, we sought to investigate which function of Ctf4 is responsible for this negative interaction. To determine if the growth defect observed in *cac1*Δ *ctf4*Δ mutant is a direct effect of uncoupling between helicase and DNA-polymerase α (Polα) [82] [83] [119], we analyzed the consequences of inactivating *CAC1* in the thermosensitive (ts) mutant *cdc17-1* encoding the catalytic subunit of Polα. We found that *cac1*Δ did not affect the viability of *cdc17-1* cells (Figure S5A), which indicates that the growth defect of *cac1*Δ *ctf4*Δ was not related to the *ctf4*Δ cell's inability to incorporate Polα into the replisome. This result shows that replication fork architecture defects, due to uncoupling arising in *ctf4*Δ mutant, are not responsible for the synthetic fitness defects observed in *cac1*Δ *ctf4*Δ cells. Moreover, the loss of Ctf4 affects DNA damage tolerance function due to faulty MCM-uncoupled Polα/Primase activity [93]. Thus, this result also indicates that the severe growth defect observed in *cac1*Δ *ctf4*Δ was not a consequence of defective DNA damage tolerance. We next searched for synthetic growth defects caused by combining *cac1*Δ mutation with the *mcm2-3A* mutation known to affect the Ctf4-Mcm2-Polα-dependent transfer of (H3-H4) parental histones [85] [120]. We found that affecting the parental histones transfer onto the lagging strand did not cause growth defects in absence of CAF-1 function (Figure S5B).

Another major function assigned to Ctf4 is associated with its role in cohesion establishment. Genetic analyses have defined two pathways for cohesion establishment at the replication fork, one containing *CTF4, CHL1, CSM3, TOF1*, and the second containing *MRC1, CTF18, CTF8, DDC1* [69]. We addressed if any of these non-essential replisome proteins, previously implicated in cohesion establishment, were required for growth in *cac1*Δ cells. We found that only *CTF4* inactivation became deleterious in absence of *CAC1* (Figure S5C-J).

These data suggest that the important growth defect observed in the *ctf4*Δ *cac1*Δ mutant was unrelated to the Ctf4 functions mentioned above.

### *CAC1* is required to sustain the viability of cells affected in essential cohesion pathways

**Figure 4:**
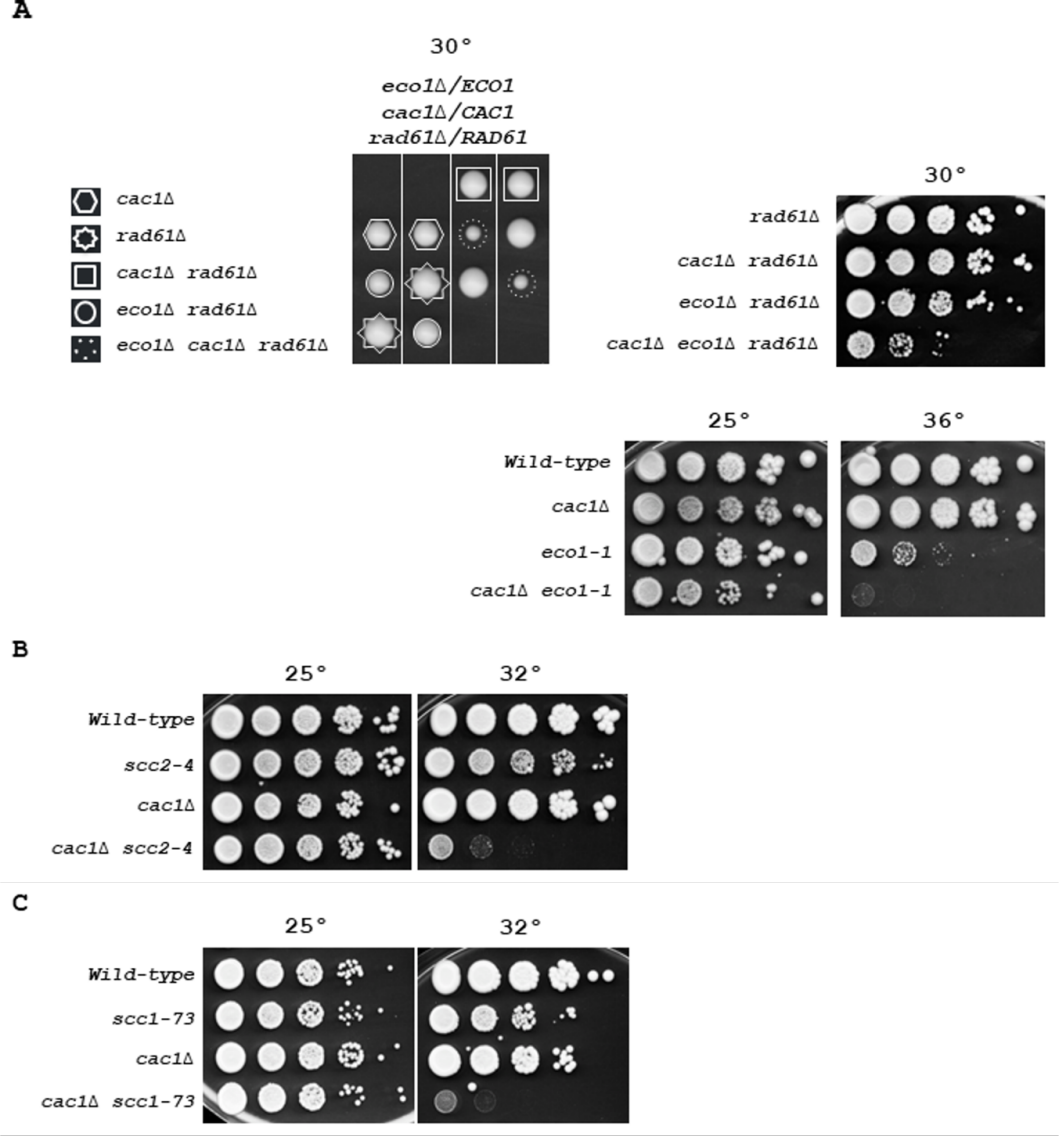
FIGURE 4: Essential sister chromatid cohesion genes are required for the viability of CAF-1 deficient cells. **(A)** Genetic interaction of *CAC1* with *ECO1*. Left, fifty tetrads from *eco1*Δ/*ECO1 cac1*Δ/*CAC1 rad61*Δ/*RAD61* diploid strain were dissected. Tetrads were grown at 30° for 5 days. Dashed circles indicate *eco1*Δ *cac1*Δ *rad61*Δ mutant. Right, top, tenfold serial dilutions of indicated genotypes were spotted onto YPD plates and incubated at 30° for 3 days. Right, down, tenfold serial dilutions of wild-type, *cac1*Δ, *eco1-1*, and *eco1-1 cac1*Δ cells were spotted onto YPD plates and incubated at 25° (left), or 36°C (right) for 3 days. **(B)** Genetic interaction of *CAC1* with *SCC2*. Tenfold serial dilutions of exponentially growing cells were spotted onto YPD plates and incubated at 25° (left), or 32° (right) for 3 days. **(C)** Genetic interaction of *CAC1* with *SCC1*. Tenfold serial dilutions of exponentially growing cells were spotted onto YPD plates and grown at 25° (left), or 32° (right) for 3 days.

Because Ctf4 is involved in multiple cohesion establishment pathways, its absence negatively influences sister chromatid cohesion in different ways [69] [77] [93] [121]. Among the non-essential proteins associated with replisomes involved in the two parallel pathways for cohesion establishment at the replication fork [69], *ctf4*Δ mutant is the only one that causes lethality in the absence of the S-phase acetyltransferase *ECO1* [121], a protein that locks sister chromatid entrapment by acetylating Smc3 both during S phase and in response to DNA damage [54] [55] [123] [124]. Therefore, we wondered whether affecting cohesion, in a more severe way than deleting non-essential cohesion genes, might reveal the importance of *CAC1* for the growth of cells affected in cohesion. We first investigated the relationship between *CAC1* and the essential cohesion genes *ECO1*. Since the inactivation of *RAD61*/*WPL1*, which counteracts cohesion-establishing reaction, suppresses *eco1*Δ lethality [54] [55], we deleted one allele of *ECO1* and one allele of *RAD61* in a *cac1*Δ/*CAC1* diploid strain. We found that *cac1*Δ *rad61*Δ cells grew normally and that *cac1*Δ cells exhibited a synthetic sick phenotype with *eco1*Δ *rad61*Δ mutations at 30°C (**Figure 4A**, left). *RAD61* inactivation impacts cohesion, chromatin structure, and intra-chromosomal loops organized by cohesins [125] [126] [127]. Thus, we also analyzed the consequence on *cac1*Δ cells of the temperature-sensitive *eco1-1* mutation which confers severe cohesion defects at 37°C, and to a lesser extent, at lower temperatures [53]. We found that the *eco1-1* mutant was strongly affected by *CAC1* inactivation (**Figure 4A**, right), which confirmed that Cac1 was required for efficient growth in *eco1* mutants. These data suggest that Cac1 could act, at least in part, in a cohesion pathway parallel to the Smc3ac function.

To further test whether in the absence of *CAC1* cohesion defects are deleterious, we combined *cac1*Δ with mutations in genes that affect the cohesin loader (Scc2-Scc4) and the cohesin ring complex (Scc1). We first analyzed the consequences of *CAC1* deletion in *scc2-4* temperature-sensitive mutant defective in cohesion establishment. Whilst at permissive temperature (25°C), *scc2-4 cac1*Δ cells were indistinguishable from *scc2-4* and *cac1*Δ, the double mutant cells were strongly affected at the semi-permissive temperature of 30°C (**Figure 4B**). We next focused on Scc1, which is involved in both establishment of cohesion in S phase and the maintenance of cohesion in G2/M. Using the temperature-sensitive *scc1-73* allele [128], we found that impairment of cohesin function strongly affects cell viability in the absence of any of CAF-1 subunits, Cac1, Cac2, or Cac3 (**Figure 4C**, and Figure S6A). Interestingly, we observed a slight difference in the severity of *cac1*Δ, *cac2*Δ, and *cac3*Δ mutant phenotypes, with the *cac3*Δ mutant showing a weaker phenotype (Figure S6A). Again, this could be explained by previous observations showing that *CAC3* inactivation has a minor effect on nucleosome formation compared to *CAC1* or *CAC2* inactivation [100]. The similar weaker phenotype observed for *cac3*Δ compared to *cac1*Δ and *cac2*Δ mutants in both *scc1-73* (Figure S6A) and *ctf4*Δ cells (**Figure 1** and Figure S2), suggests that the requirement for Cac1 in *ctf4*Δ cells is related to important cohesion defects linked to the absence of Ctf4. *cac1-F233L* and *cac1-F233A,F234G* mutations were previously shown to alter Cac1 binding to PCNA as well as Cac1 DNA-replication-linked nucleosome assembly function [17]. To investigate the importance of Cac1 recruitment to the replication forks through its interaction with PCNA, we determined the impact of *cac1-F233L* and *cac1-F233A, F234G* mutations in *scc1-73* cells. We found that both mutations caused growth defects for *scc1-73* cells at permissive temperature (Figure S6B). These data indicate that the PCNA-dependent CAF-1 recruitment to chromatin is required when cohesin function is affected. These results confirm that CAF-1 complex is required for cell viability when cohesion is affected. On the other hand, CAF-1 and Rtt106 function in a coordinated manner in nucleosome assembly [107]. Henceforth, we tested whether *RTT106* deletion affects the growth of the *scc1-73* mutant. A significant growth defect was observed in *rtt106*Δ *scc1-73* cells at the semi-permissive temperature compared to each *rtt106*Δ and *scc1-73* single mutant (Figure S6C). In agreement with the fact that *RTT106* exerts a minor role compared to CAF-1 in new histones deposition during DNA synthesis [26], we found that the growth defect of the *rtt106*Δ *scc1-73* mutant was much weaker than that of the *cac1*Δ *scc1-73* mutant. Furthermore, *cac1*Δ *rtt106*Δ *scc1-73* triple mutant exhibited a more dramatic effect compared to the *rtt106*Δ *scc1-73* and *cac1*Δ *scc1-73* double mutants (Figure S6C). Taken together these results indicate that CAF-1, and more generally nucleosome assembly during replication, is crucial in maintaining genome stability when SCC is compromised. These results, in association with our previous findings showing that *cac1*Δ *ctf4*Δ cells are more affected than *rtt106*Δ *ctf4*Δ cells and that *RTT106* inactivation amplified the growth defect of *cac1*Δ *ctf4*Δ cells (**Figure 1A**), strongly suggest that the requirement for CAF-1 in *ctf4*Δ mutant was related to important cohesion defects linked to the absence of *CTF4*.

**Figure 5:**
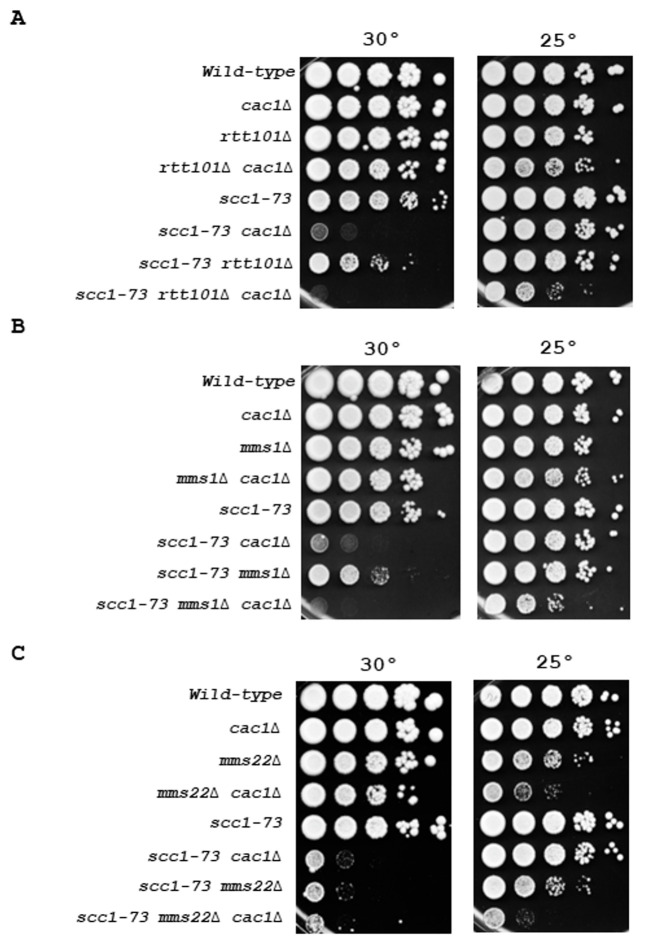
FIGURE 5: Cac1 functions in a different pathway from that of Rtt101-Mms1-Mms22 in cohesion. Genetic interactions among *RTT101, MMS1, MMS22, SCC1*, and *CAC1*. **(A)** Effect associated with *RTT101* inactivation on viability of *cac1*Δ, *scc1-73*, and *cac1*Δ *scc1-73* cells. **(B)** Effect associated with *MMS1* inactivation on viability of *cac1*Δ, *scc1-73*, and *cac1*Δ *scc1-73* cells cells. **(C)** Effect associated with *MMS22* inactivation on the viability of *cac1*Δ, *scc1-73*, and *cac1*Δ *scc1-73* cells. Genetic interactions were assessed by spotting a tenfold series dilution of cells of the indicated genotype onto YPD. Cells were grown at 30° for 3 days (left), or at 25°C (right) for 4 days.

Ctf4 interacts with Mms22 [82] [84] [90], an adaptor protein of the Rtt101-Mms1 E3 ubiquitin ligase complex to tether the Rtt101-Mms1-Mms22 E3 ubiquitin ligase to active replisome during S-phase [86]. Previous studies showed that Rtt101-Mms1-Mms22 inactivation is epistatic with *ctf4*Δ regarding cohesion defects [37]. We first assessed the importance of the ubiquitin ligase in *ctf4*Δ cells and found that its absence did not significantly affect growth (Figure S7). We next addressed the consequences of deleting *RTT101, MMS1*, or *MMS22*, in *cac1*Δ, *scc1-73*, and *cac1*Δ *scc1-73* cells. We found that at 30°C, the growth of *cac1*Δ *rtt101*Δ, *cac1*Δ *mms1*Δ, and *cac1*Δ mms22 double mutants was not strongly affected compared to one of the *rtt101*Δ, *mms1*Δ, and *mm22*Δ single mutants (**Figure 5A, B, C**, left). These results strongly suggest that sister chromatid cohesion established through the interaction between Rtt101-Mms1-Mms22 E3 ubiquitin ligase and Ctf4 has little or no involvement in the growth defect observed for *cac1*Δ *ctf4*Δ cells. Moreover, we found that impairment of cohesin function dramatically affected *cac1*Δ viability compared to *rtt101*Δ or *mms1*Δ (**Figure 5A, B**, left). We observed a higher impact for *MMS22* inactivation compared to *RTT101* or *MMS1* inactivation. This may be explained by the multiple roles of Mms22 in response to DNA damage [31] [92] [96] and/or by an Mms22 role in promoting cohesion through its direct interaction with Eco1 [37] [39]. Interestingly, at the permissive temperature for the *scc1-73* mutant (25°C, right), we observed that *scc1-73 cac1*Δ *rtt101*Δ, *scc1-73 cac1*Δ *mms1*Δ, and *scc1-73 cac1*Δ *mms22*Δ cells are more affected than each of the double mutants, suggesting that CAF-1 could act in parallel with the Scc1 and Rtt101-Mms1-Mms22 cohesion pathways. Taken together these genetic analyses indicate that the growth defect of *cac1*Δ *ctf4*Δ cells is not the consequence of cohesion defects caused by a deficiency in the Rtt101-Mms1-Mms22-Ctf4 replication-coupled sister chromatid cohesion pathway, but rely on another pathway independent of the Rtt101-Mms1-Mms22 E3 ubiquitin ligase complex.

### Cac1 is required for sister chromatid cohesion in *ctf4*Δ cells

**Figure 6:**
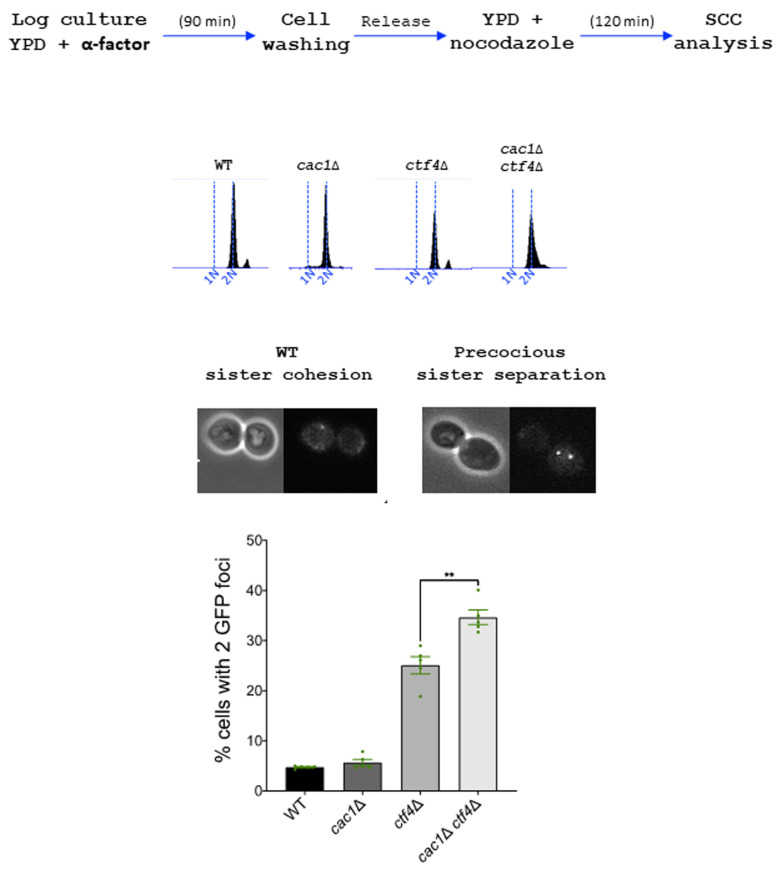
FIGURE 6: Cac1 is important to maintain cohesion in *ctf4*Δ cells. Sister chromatid cohesion was analyzed by monitoring the tagged centromere of chromosome III. Top, experimental design. DNA replication was monitored by FACS analysis of DNA content. Over 100 cells were counted for each experiment. The results represent the average of five independent experiments. Statistical significance was measured using Two Tailed Mann-Whitney test.

In contrast to *ctf4*Δ cells, which present important cohesion defects [129], *cac1*Δ cells exhibit almost none or only moderate cohesion defects according to previous studies [37] [121]. To address whether the synthetic growth defect of *cac1*Δ *ctf4*Δ cells could be due to an additive effect on sister chromatid cohesion, we compared cohesion in metaphase-arrested *cac1*Δ and *ctf4*Δ cells to cohesion in *cac1*Δ *ctf4*Δ double mutant. To evaluate sister chromatid cohesion, we used a haploid strain containing Lac operator tandem repeats integrated at a site near the centromere of chromosome III and expressing a GFP-Lac repressor fusion protein. We found that *ctf4*Δ cells exhibited cohesion defects and that cohesion was not significantly affected by the absence of CAF-1, suggesting that CAF-1 is not important for cohesion in a wild-type context. However, cohesion defects in the *cac1*Δ *ctf4*Δ double mutant were significantly more severe than that in *ctf4*Δ single mutant (**Figure 6**). While we do not know the reason behind this increased cohesion defect, this result together with our genetic analyses, strongly suggests that, when cohesion is severely affected, CAF-1 exerts an important role required for cohesion maintenance and cell viability.

### Cohesin association to chromatin is increased in the absence of Cac1 in WT and *ctf4*Δ cells

**Figure 7:**
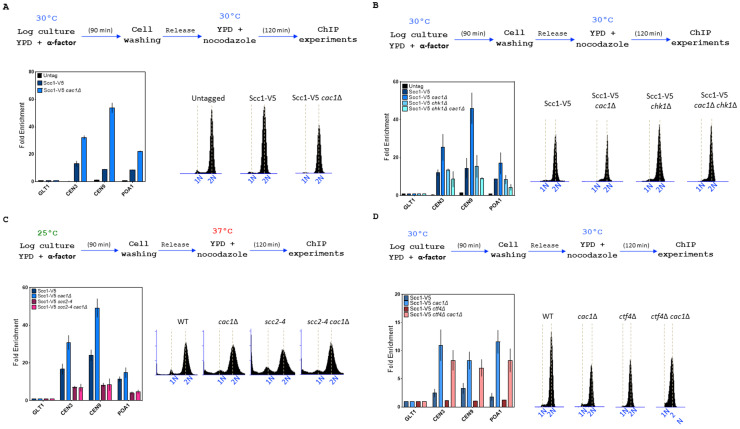
FIGURE 7: *CAC1* inactivation increases cohesin level at chromatin in WT and *ctf4*Δ cells. Top, experimental design. In all experiments, DNA replication was monitored by FACS analysis of DNA content. **(A)** *CAC1* inactivation increases cohesin level at centromere and chromosome arm. Cells of the indicated genotype were synchronized in G1 and were released into nocodazole imposed mitotic arrest for 120 minutes. Scc1 levels at two centromeres (*CEN3* and *CEN9*), a chromosome arm cohesin binding site (*POA1*), and a negative control-binding site (*GLT1*) were measured by ChIP, followed by real-time qPCR. SEM shown represents four independent experiments. **(B)** The increased cohesin level at chromatin observed in absence of *CAC1* is due to DNA damage. ChIP-qPCR analyses of Scc1 level at centromere and chromosome arm in WT, *cac1*Δ, *chk1*Δ, and *chk1*Δ *cac1*Δ. Same experimental conditions as in (A). **(C)** ChIP-qPCR analyses of Scc1 level at centromere and chromosome arm in WT, *scc2-4, cac1*Δ, and *cac1*Δ *scc2-4*. **(D)** *CAC1* inactivation increases cohesin level at centromere and chromosome arm in absence of *CTF4*.

We next investigated if the level of Scc1, at known cohesin binding sites on chromatin, was affected in the absence of CAF-1 function by performing ChIP experiments in WT and *cac1*Δ cells. To our surprise, we found that deletion of *CAC1* markedly increased cohesin levels both at centromeres and chromosome arms (**Figure 7A**). Cohesin enrichment is enhanced genome-wide in response to DSB induction and this enrichment at undamaged sites globally tight-ens sister chromatid cohesion [124] [130] [131]. Taking into account that CAF-1 plays multiple roles in maintaining genome stability [3] [6] [7] [11] [13] [96] [98] [122] and that the absence of Cac1 in both wild-type and *ctf4*Δ cells increased Rad52 foci (**Figure 3A**, left), Rfa1 foci (**Figure 3A**, right) [7], and γH2AX (**Figure 3B**), a possible explanation could be that DNA-damage-induced cohesion establishment was the source of the high cohesin level arising in absence of Cac1. Because Chk1, which mediates the DNA damage response in parallel with *RAD53*, is a key component of the damage-induced cohesion establishment pathway required for the generation of damage-induced cohesion [134], we analyzed cohesin levels in absence of Chk1 in WT and *cac1*Δ cells. We observed that the cohesin levels were indistinguishable between *chk1*Δ and wild-type cells and found that deletion of *CHK1* in *cac1*Δ mutant reduced the association of Scc1 at both centromeres (CEN3, CEN9) and chromosome arms (PAO1) (**Figure 7B**). This indicates that DNA damage-induced cohesion establishment is the source of the increased Scc1 level observed in absence of *CAC1*.

Cohesin association with centromeres, promoters, DSBs, and stalled replication forks depends on the Scc2-Scc4 complex [41] [45] [135] [136]. To determine whether the Scc2-Scc4 complex was involved in the higher level of cohesins observed in *cac1*Δ cells, we used the *scc2-4* thermosensitive mutant and compared the level of Scc1 at centromeres and chromosome arms in wild-type, *scc2-4, cac1*Δ and *cac1*Δ *scc2-4* cells. To this end, we synchronized cells by α-factor pheromone block, released them into S-phase at 25°C, and subsequently cultivate them at the restrictive temperature for the *scc2-4* mutation in the presence of nocodazole to arrest cells in G2/M. As expected, at restrictive temperatures, cohesin levels were strongly decreased in *scc2-4* mutant compared to wild-type cells (**Figure 7C**). Interestingly, our chromatin immunoprecipitation experiments revealed similar cohesin levels both at centromeres and chromosome arms of *scc2-4* and *scc2-4 cac1*Δ cells, revealing that the increased level of cohesin association observed in *cac1*Δ cells depends on the cohesin loader Scc2 (**Figure 7C**).

We further compared the level of Scc1 on chromatin in WT, *cac1*Δ, *ctf4*Δ, and *cac1*Δ *ctf4*Δ cells. As expected we found that Scc1 binding at chromatin was greatly reduced in *ctf4*Δ cells [77] [121]. Compared to *cac1*Δ cells, *cac1*Δ *ctf4*Δ cells exhibited a slight reduction in Scc1 binding, which interestingly, remained much higher than in *ctf4*Δ mutant and WT (**Figure 7D**). The fact that Scc1 occupancy is higher in *cac1*Δ *ctf4*Δ double mutant compared to *ctf4*Δ single mutant while the double mutant exhibits a stronger cohesion defect, suggests that cohesins are not fully functional in *cac1*Δ *ctf4*Δ cells, possibly because they are not able to reach an efficient cohesive state when *ctf4*Δ mutation is combined with *CAC1* gene deletion.

### Cac1 is required for the efficient acetylation of Smc3

**Figure 8:**
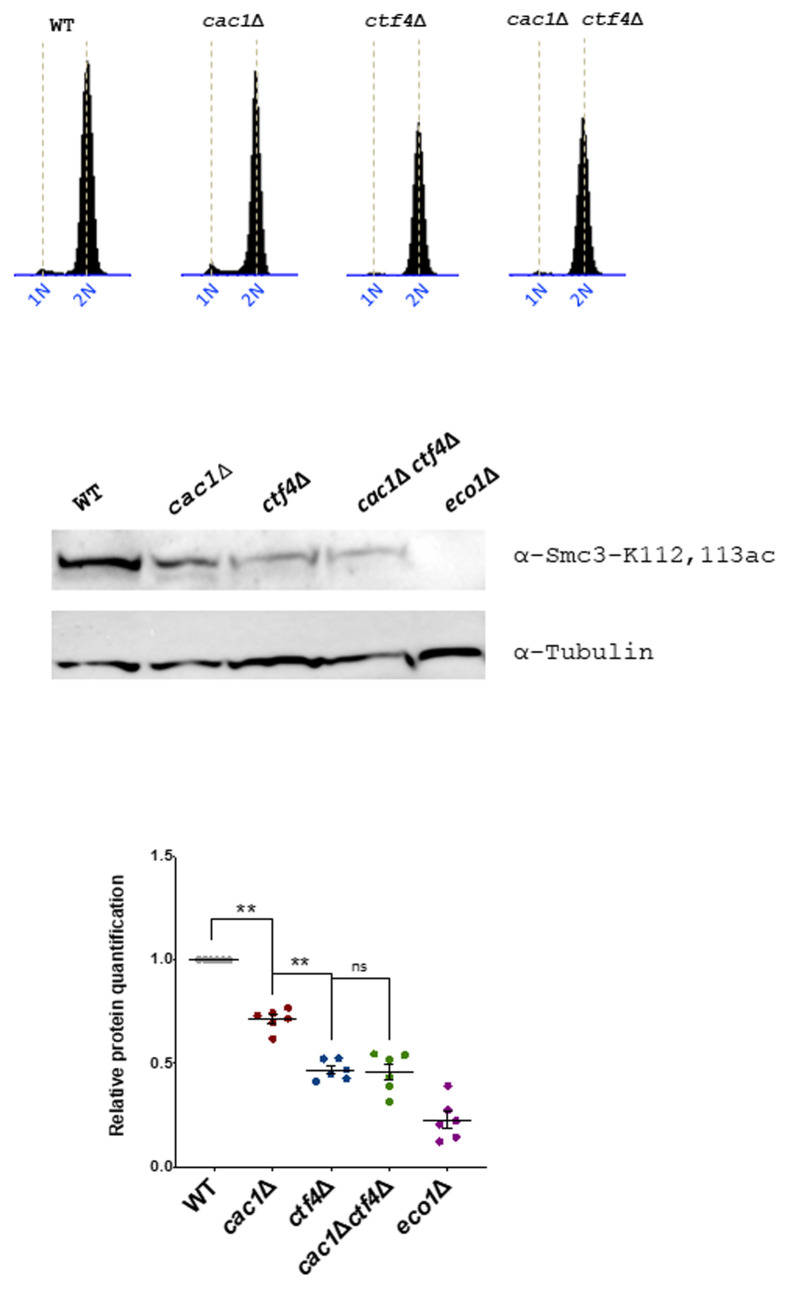
FIGURE 8: Eco1-catalyzed Smc3 acetylation is reduced in the absence of Cac1. Cells of the indicated genotypes were synchronized in G1 using α-factor and released into nocodazole-imposed mitotic arrest at 25°. Top, FACS analysis of the DNA content was used to monitor cell cycle progression. Middle, acetylated Smc3 was immunoblotted in the cell lysate by an antibody specific to Smc3-K112,113Ac (gift from Adele Marston, The Wellcome Centre for Cell Biology, Edinburgh, UK). Down, quantitative Western blotting analyses of Smc3-K112,113 acetylation. The results represented the average of six independent experiments. Statistical significance was measured using Two Tailed Mann-Whitney test.

Cohesion establishment mainly depends on Eco1 which acetylates the Smc3 cohesin subunit at lysine 112 (K112) and 113 (K113) both during S phase and independently of DNA replication [52] [53] [54] [137] [138]. To further understand the genetic relationship between *CAC1* and *CTF4* related to cohesion, we investigate whether CAF-1 contributed to Smc3ac in WT and *ctf4*Δ cells. We analyzed Smc3ac after a nocodazole-imposed mitotic arrest by Western blot using a validated antibody. As expected from previous data [121], we observed that the Smc3-K112,113 Eco1-dependent acetylation is strongly diminished in *ctf4*Δ cells. Our quantitative Western blotting of Smc3-K112,113 acetylation (Smc3-K112,113ac) showed that *cac1*Δ mutation did not amplify the Smc3-K112,113ac defect observed in absence of Ctf4 but interestingly, revealed that CAF-1 deficient cells displayed partial loss of Smc3-K112,113ac (**Figure 8**). Because *CAC1* is required for SCC in *ctf4*Δ mutant (**Figure 6**), this suggests that CAF-1 acts, at least in part, in a pathway parallel to Smc3ac to promote cohesion.

## DISCUSSION

H3K56 acetylation is a histone mark required for genome stability maintenance during replication stress and for chromatin assembly during replication (reviewed in [139]). In a previous study, we found that replisome function is modulated during replicative stress by H3K56ac through an interaction between Rtt101-Mms1-Mms22 and Ctf4. We revealed that Ctf4 became deleterious under replication stress in the absence of the DNA repair/tolerance branch of the H3K56ac pathway [92] but left unanswered the importance of Ctf4 when the nucleosome assembly branch of the H3K56ac pathway is affected during replication. Here we show that mutants lacking any subunit of the chromatin assembly factor CAF-1 (Cac1, Cac2, Cac3) exhibit synthetic sickness in absence of *CTF4* gene, suggesting that Cac1 and Ctf4 jointly participate in an essential process of the cells. To uncover this essential process, we performed genetic analyses. We observed that *ctf4*Δ is deleterious when combined with mutations affecting various histone chaperones known to be involved in replication-coupled chromatin assembly, and with mutations at H3 or H4 histone lysine residues implicated in nucleosome assembly during replication. On the contrary, inactivation of the HIR complex, which is involved in replication-independent nucleosome assembly, as well as deletion of *NAP1*, encoding for a histone chaperone involved in H2A and H2B histones deposition, does not induce a growth defect in *ctf4*Δ cells. These data highlight the crucial role of replication-coupled chromatin assembly in absence of *CTF4*. We further show that *asf1*Δ and *rtt109*Δ deletions caused only a modest synthetic sickness with *ctf4*Δ (compared to *cac1*Δ, *cac2*Δ or *cac3*Δ) even though they abrogate the H3K56ac pathways. Along the same line, the *ctf4*Δ mutant is only slightly affected by the absence of the Rtt101-Mms1-Mms22 E3 ubiquitin ligase complex which functions with Asf1 and Rtt109 in the H3K56ac pathways [25] [92]. We infer that this difference reflects the much more important role exerted by CAF-1 in chromatin assembly during replication. This assumption is reinforced by previous data showing that mutating the Rtt101-Mms1-Mms22 complex affects the association of H3-H4 with the histone chaperones Asf1 and Rtt106 but does not alter the binding of H3-H4 to CAF-1 [25], suggesting that H3 ubiquitination promotes the transfer of H3-H4 from Asf1 to Rtt106 but not to CAF-1. Moreover, nucleosome assembly proceeds in an ordered manner in the absence of Asf1, Rtt109, and H3K56ac, but appears to be severely disrupted upon deletion of *CAF-1* [140]. Thus, we propose that the H3K56ac-dependent DNA repair/tolerance mechanisms (that do not require CAF-1), as well as the H3K56ac-chromatin assembly function of Rtt101-Mms1-Mms22, are not important for the growth of *ctf4*Δ mutant contrary to CAF-1-dependent replication-coupled nucleosome assembly function whose mutation leads to synthetic sickness in *ctf4*Δ cells. Taken together, our data strongly suggest that the synthetic sickness observed for CAF-1 deficient cells in absence of *CTF4* is a direct consequence of nucleosome assembly defects arising during replication.

We show that the combined absence of *CAC1* and *CTF4* is lethal for cells experiencing exogenous DNA damage and that Rrm3, whose function is to assist fork progression across pausing sites, is essential for the viability of *cac1*Δ *ctf4*Δ cells. In addition, we show that both the S-phase and the spindle-assembly checkpoints are required for *cac1*Δ *ctf4*Δ viability. We also report a much higher frequency of spontaneously arising Rfa1 and Rad52 foci, an increased level of γH2AX, as well as an increased level of spontaneous mutation rate in *cac1*Δ *ctf4*Δ cells compared to every single mutant and wild-type cells. Therefore, we conclude that the absence of CAF-1 function causes an important genetic instability in *ctf4*Δ cells leading to the emergence of toxic DNA structures and/or DSB during replication, that needs to be repaired by homologous recombination.

Ctf4 has been implicated in multiple chromosomal functions [77] [81] [82] [84] [85] [92] [93] [119] [141] [142]. Our genetic analysis conducted to uncover which function of Ctf4 is crucial in absence of *CAC1* reveals that the functions of Ctf4, in maintaining normal replisome architecture, in promoting the coordination of leading and lagging strands during replication, and in DNA damage tolerance, are not responsible for the sickness observed for *cac1*Δ *ctf4*Δ cells. In addition, contrary to DNA-replication coupled nucleosome assembly, the transfer of parental histones to lagging strands during replication promoted by Ctf4 is not required for the growth of *cac1*Δ *ctf4*Δ cells. These findings suggest that the problem arising in *cac1*Δ *ctf4*Δ mutant lies in the deposition, during replication fork progression, of H3-H4 histones synthesized *de novo* rather than in recycling parental histones.

**Figure 9:**
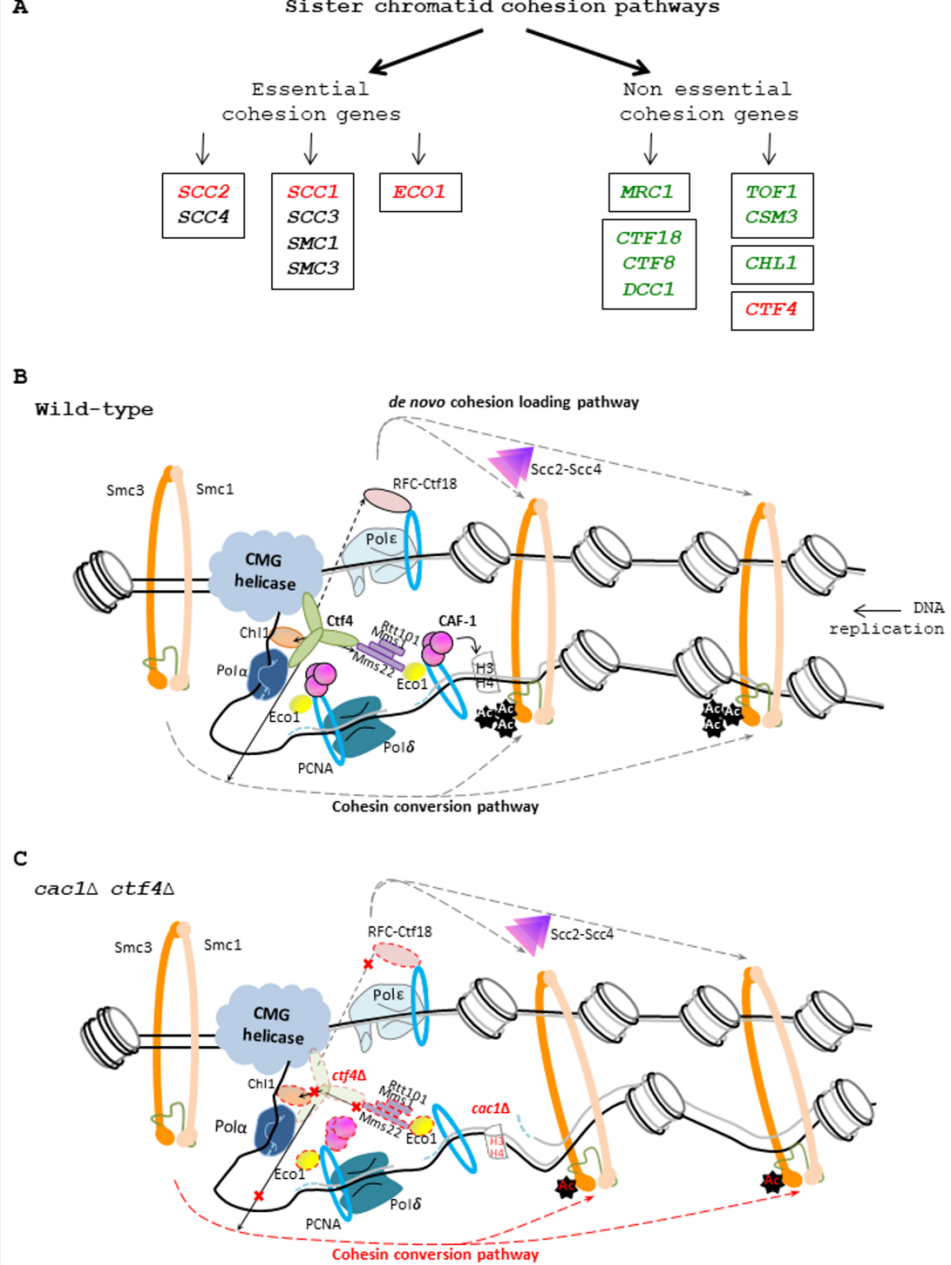
FIGURE 9: CAF-1 and the replisomal protein Ctf4 are important for cohesion. **(A)** Genetic interactions between CAF-1 complex and genes involved in sister chromatid cohesion. Left, essential cohesion genes. Right, non-essential cohesion genes encoding replisome-associated proteins that are essential for efficient Smc3 acetylation by Eco1, and for cohesion establishment [69] [121]. The Mrc1, CTF18-RFC pathway is involved in *de novo* loading of cohesins onto nascent DNAs [74]. The Tof1/Csm3, Chl1, Ctf4 pathway is involved in conversion of chromosome associated cohesins into cohesive structure during S phase [74]. Genes enclosed encode protein to form a complex. Genes in green are not required for growth in absence of CAF-1 function. Genes in red are required for growth in absence of CAF-1 function. For essential genes, we used thermosensitive mutants to analyze the genetic interactions with CAF-1 mutants. Genes in black: Genetic interaction with CAF-1 mutants non-determined. **(B)** Illustration of the role played by Ctf4 in the SCC establishment. Ctf4 links DNA replication with sister chromatid cohesion establishment by recruiting Chl1 helicase to the replisome where it directly interacts with cohesins and assists Eco1, which acetylates Smc3, in replication coupled-cohesin establishment [38] [77]. Ctf4 interacts and tethers the Rtt101-Mms1-Mms22 E3 ubiquitin ligase to the replisome which in turn recruits and/or promotes Eco1-dependent sister chromatid cohesion [37] [86]. Ctf4 can also help the chromatin recruitment of Ctf18-RFC [56] a complex that, loads and unloads PCNA, could act as a binding platform for recruiting Eco1, and is involved with the cohesin loader Scc2-Scc4 in the *de no*vo loading of cohesins onto nascent DNA [74]. Finally, Ctf4 is also required for the conversion of cohesins rings preloaded onto the DNA template into a cohesive form [74]. **(C)** Proposed model explaining the importance of CAF-1 in *ctf4*Δ cells. In absence of Ctf4, multiple cohesion establishment pathways are affected (red cross) leading to major defects in SCC establishment. In absence of CAF-1 fewer nucleosomes are deposited on replicated DNA, generating fewer but longer Okazaki fragments [140], leading to increased inter-nucleosome spacing in nascent chromatin and inappropriate epigenetic states [13] [148]. In these conditions, the nucleosome assembly function of CAF-1 is required to maintain SCC and efficient cell growth in yeast affected in cohesion. In *ctf4*Δ *cac1*Δ cells, the altered chromatin structure arising in absence of CAF-1 function increases the SCC defects induced by the absence of Ctf4, leading to severe growth defects and genomic instability.

Ctf4 is also required for cohesion, which is established at the time of replication. Two parallel pathways for cohesion establishment at the replication fork, involving non-essential genes encoding for proteins associated with replisomes, one containing *CSM3, TOF1, CTF4*, and *CHL1*, and the second containing *MRC1, CTF18, CTF8*, and DCC1, have been defined [69]. Our genetic analyses revealed that, among the different genes involved in these parallel pathways, *CTF4* is the only gene required for the growth of *cac1*Δ mutant. We also found that the E3 ubiquitin ligase complex Rtt101-Mms1-Mms22, known to interact and function with Ctf4 in sister chromatid cohesion [37], is not required for the growth of *cac1*Δ. At first sight, these results suggest that the defective process leading to *cac1*Δ *ctf4*Δ sickness is unrelated to Ctf4 function in cohesion. Ctf4 physically interacts and recruits Chl1 to the replisome which in turn interacts with cohesin to promote cohesion [77]. Interestingly, unlike *CHL1* deletion, and unlike deletion of genes encoding for the other establishment factors, *CTF4* deletion is lethal in *eco1*Δ (*rad61*Δ/*wpl1*Δ) mutant cells [121] that are strongly affected in Smc3-K112,113ac and as a consequence severely defective for sister chromatid cohesion [55]. This suggests that Ctf4 also acts, at least in part, in a pathway parallel to Smc3ac and independently of the Chl1 pathway. Thus, we reasoned that Ctf4 function in SCC is more important than the one exercised by each of the other proteins encoded by the non-essential genes involved in SCC. In that case, the deficient cohesion function of *ctf4*Δ mutant could be responsible for the sickness observed during the combined absence of *CAC1* and *CTF4*. Indeed, cell fractionation experiments have suggested that Ctf4 helps the chromatin recruitment of Ctf18-RFC [56], a complex involved in the *de novo* loading of cohesin onto nascent DNAs through a *CTF4*-independent pathway required for normal cohesion and cell viability in the absence of Ctf4 [74]. Moreover, Ctf4 also recruits the Rtt101-Mms1-Mms22 complex to the replisome through its interaction with Mms22, a protein that promotes Eco1 recruitment at the DNA replication fork and subsequently cohesion [37]. Thus, because Ctf4 plays a major role in cohesion establishment through multiple pathways, we propose that *cac1*Δ *ctf4*Δ sickness is a direct consequence of a role for CAF-1 function that is revealed when cohesion is affected. Such hypothesis is strongly reinforced by our findings revealing that *CAC1* inactivation increases the cohesion defect of *ctf4*Δ cells, and by the strong negative synthetic genetic interactions detected between *caf-1* mutants and mutations affecting essential cohesion genes (**Figure 9A, B**). Our findings show that mutations abolishing H3K56ac (*asf1*Δ and *rtt109*Δ) or Rtt101-Mms1-Mms22 E3 complex function (*rtt101*Δ, *mms1*Δ, or *mms22*Δ) do not significantly affect the growth of *ctf4*Δ cells contrary to CAF-1 mutations and mutations at genes playing a key role in DNA-replication coupled nucleosome assembly. We thus assume that CAF-1 function in cohesion is independent of the H3K56ac-Rtt101-Mms1-Mms22-Ctf4-dependent pathway previously described by Zhang and colleagues [37]. This pathway is known to protect against replicative damage by DNA repair/tolerance mechanisms, in a CAF-1-independent manner [27] [92]. Moreover, based on co-precipitation analyses showing that the Rtt101-Mms1-Mms22-dependent H3 ubiquitination affects H3-H4 association with Asf1 and Rtt106 but not with CAF-1 [25], our data showing that CAF-1 inactivation affects *scc1-73* cohesin mutant viability much more strongly than Rtt101-Mms1 E3 ubiquitin ligase inactivation, led us to propose that the chromatin function of CAF-1 required for cell viability in presence of SCC defects is independent of H3K56ac and Rtt101-Mms1-Mms22.

We further report that deleting *CAC1* increases the level of Scc1 on chromatin. Consistent with the observation that *cac1*Δ cells exhibit higher levels of Rfa1 spontaneous foci, of Rad52 spontaneous foci, and of γH2AX which recruits the cohesin loader at DNA double-strand breaks [143], we found that the increased Scc1 level observed in the *cac1*Δ mutant requires an intact DNA damage response. Thus, it is possible that the increased Scc1 level, induced in response to DNA damage observed in *cac1*Δ cells, is sufficient to maintain a wild-type level of cohesion. This may explain, in agreement with previous work [121], the absence of SCC defect that we observed in CAF-1 deficient cells. Indeed, we observed that deleting *CAC1* leads to a WT level of Scc1 both at centromeres and chromosome arms in the absence of the cohesin loader complex Scc2-Scc4. Because both cohesin loading and cohesin translocation on chromatin depend mainly on Scc2 and Scc4 proteins [144] [145] it is possible that the abnormal level of cohesin observed in the absence of Cac1 is the consequence of an abnormal cohesin redistribution in response to DNA damage. Nucleosomes inhibit cohesin loading [42] and it has been shown that fewer nucleosomes are deposited on replicated DNA in CAF-1 deficient cells [146]. Thus, it is possible that the temporal delay in nucleosome assembly and the increased inter-nucleosome spacing in nascent chromatin arising in CAF-1 mutants [147] [148] alter chromatin structure and cause replicative DNA damages that facilitates cohesin loading.

We further found that deleting *CAC1* in *ctf4*Δ mutant also increased Scc1 level on chromatin. How could CAF-1 inactivation in *ctf4*Δ cells simultaneously increase cohesin levels on chromatin and cohesion defects? Ctf4 is essential for converting cohesin associated with un-replicated DNA into functional cohesive structures [74] and to recruit Mms22 to the replisome [86], a protein that in turn, can recruits Eco1 at the DNA replication fork [37] [39]. A simple explanation could be that, although the level of cohesin increases in *cac1*Δ *ctf4*Δ mutant compared to *ctf4*Δ mutant, the level of functional cohesins is reduced in *cac1*Δ *ctf4*Δ mutant due to CAF-1 inactivation.

What could be the function of CAF-1 in cohesion? We do not know whether the observed effects are only the consequence of nucleosome formation defects during replication or if CAF-1 plays a direct role in SCC. So far, no physical interaction between CAF-1 and any cohesin establishment/maintenance factors nor cohesin proteins has been identified. However, it has been shown that the over-expression of CAF-1 subunits can suppress the non-viability of temperature sensitivity of *eco1* mutant at restrictive temperatures [37], and we have shown that CAF-1 inactivation negatively affects Smc3-K112,113ac, indicating that CAF-1 can influence cohesin acetylation at the replication fork during S phase, and suggesting that CAF-1 could function directly in cohesion establishment. CAF-1 and Eco1 both interact with PCNA through a PIP-box, and this interaction is crucial for Eco1 to promote cohesion establishment as well as for CAF-1 to sustain the viability of cohesin mutants. One possibility is that CAF-1 facilitates the recruitment and/or the stabilization of the acetyltransferase Eco1 at the replication fork. It was recently nicely shown that two transient DNA structures that form during Okazaki fragment maturation promotes cohesin acetylation to stabilize newly established sister chromatid cohesion [58]. Okazaki fragments processing and nucleosome assembly are interlinked [140] [147]. Depletion of CAF-1, but not the absence of H3K56ac, completely ablates the nucleosome-sized periodicity of Okazaki fragments, and generates fewer but longer Okazaki fragments [140]. It is possible that the lower nucleosome density and the longer Okazaki fragments generated in the absence of CAF-1 affect the surrounding and/or the access of proteins required for cohesion, leading to a defective Smc3ac and to cohesion defects.

Our genetic pieces of evidence argue that the CAF-1-related defects in SCC are additive with those arising from improper loading of cohesin (*scc2-4* mutant), from improper stabilization of cohesin (*eco1* and *scc1-73* mutants), and improper activation of cohesin (*ctf4*Δ mutant). This favors the idea that CAF-1 is involved in SCC through a cohesin-independent pathway that is additive with the cohesin-mediated pairing pathways. CAF-1 could participate in the maintenance of cohesion through its capacity to create a chromatin structure that maintains sister chromatids in proximity when cohesion is affected. If so, this chromatin structure mediated by CAF-1 should be established during fork progression because we have shown that the interaction between CAF-1 and PCNA, which recruits CAF-1 at replication forks, is crucial for the viability of *scc1-73* mutant in the presence of cohesion defects. Disruption of the interaction between PCNA and CAF-1 causes silencing defects [14] [16]. CAF-1 contributes to the maintenance of silencing independently of Asf1 (and H3K56ac) at the transcriptionally silent *HML* loci [149] and is important to ensure the inheritance of the appropriate epigenetic state [12] [13]. CAF-1-mediated chromatin structure may help the recruitment of a specific factor not directly involved in cohesion, but that favors cohesion. Cac1 directly interacts with the heterochromatin protein Sir1 which interacts with the origin recognition complex ORC [150] [151] and contributes to silencing. Interestingly, ORC mutants do not exhibit any cohesion defects by themselves but genetic analyses revealed an interaction between the ORC genes and SCC genes [152] [153] [154]. ORC is involved in Smc3ac [153], and similarly to *caf-1* mutations, *orc* mutations are additive with *eco1-1* and cohesin defects [152] [153]. Thus, it may be that CAF-1 similarly to ORC maintains a global chromatin structure that is not important for cohesion establishment and maintenance but is required to maintain sister chromatids in proximity in the presence of important cohesion defects (**Figure 9C**).

In summary, we have demonstrated that CAF-1 (but not H3K56ac) is crucial to guard genome stability in the absence of the replisomal protein Ctf4. We show that yeast lacking both *CAC1* and *CTF4* present an increased mutation rate, and require the S-phase and the spindle checkpoint pathways as well as HR to survive, revealing the presence of important damages. Furthermore, our detailed genetic analyses demonstrate that CAF-1 is required for cell growth in the presence of SCC defects and highlight the major role played by Ctf4 in cohesion. We also point out that the absence of CAF-1 increases the level of cohesin on chromatin and reduces cohesin acetylation. This work reveals novel roles for CAF1 related to its nucleosome assembly function, in the maintenance of genome stability.

## MATERIALS AND METHODS

### Strain construction

All strains used in this study are presented in Table S1. To obtain gene deletions we amplified by PCR a disruption cassette containing the appropriate marker, as described previously [155].

### Spore viability and growth

Diploid strains were sporulated at 25°C during 3 days on solid sporulation media and treated with 3 μl of 1 mg/ml zymolyase 20T (Seikagaku Biobusiness, Japan) during 10 minutes in water before tetrads were dissected on rich media (YPD plate). We used a MSN400 micromanipulator from SINGER Instruments. Viable colonies were scored 3 days or 5 days later. An average of 50 tetrads were dissected. The number of tetrads analyzed is denoted in the figure legends. Quantification of the spore growth was done by image analysis of area of growth using Image J.

### Fluorescence microscopy

Microscopy analyses were carried out in liquid media supplemented in adenine using a Nikon Eclipse Ti microscope with a 100x objective. We used a Neo sCMOS camera (Andor) to collect the images. The exposure time was DIC: 500 ms; CFP: 75 ms, YFP: 75 ms, and GFP 75 ms. We used ImageJ to analyze the images on 2D-maximum projections from 11-Z-stacks spaced 0.5 μ each. All the cells analyzed were prepared by growing the cells at 30°C in YPD media supplemented in adenine.

### Mutation rate analysis

Yeast cells were grown overnight in liquid YPD medium at 30°C. Cells were then diluted to 0.2 OD_600nm_ in 1 ml of water. Tenfold serial dilutions were done in water and each dilution was plated on YPGal plates to induced *TUS* expression and obtain separated colonies after 3 days growth at 30°C. YPGal plates were then replica plated twice onto 5-FOA plates to confirm the 5-FOA resistant phenotype of the growing colonies. Mutation rates were measured by fluctuation analysis [156] [157]. Statistical analyses were done on n=5 independent experiments using two-tailed Mann-Whitney test.

### Chromatin Immunoprecipitation (ChIP)

We used the following steps to prepare chromatin samples: We first crosslinked the cells for 15 minutes with formaldehyde (1%) and used glycine (125 mM) to quench the reaction for 5 minutes. Cells were then lysed in 50 mM HEPES-KOH [pH7.5], 140 mM NaCl, 1 mM EDTA, 1% Triton X-100, 0.1% sodium deoxycholate (FA lysis buffer) supplemented with cocktail of protease inhibitors by vortexing cells with glass beads (6 cycles × 20 s, with cooling between the cycles). After a centrifugation to remove debris, we then used a Bioruptor Pico sonicator to share the chromatin to around 200 bp. Insoluble materials were removed by centrifugation for 10 minutes (14,000 rpm, 4 °C). We mixed 500 μg of chromatin and the recommended amount of anti-V5 and conducted the immunoprecipitation overnight. We next added 25 μl of FA lysis buffer containing Protein G-Sepharose beads (3 hours incubation at 4 °C). Protein G-Sepharose beads were next washed successively once with FA buffer and twice with a FA buffer containing 500 mM NaCl. We next washed the protein G-Sepharose beads two times using a wash buffer composed of 10 mM Tris-HCl [pH 8.0], 0.25 M LiCl, 1 mM EDTA, 0.5% NP-40, 0.5% Na-Deoxycholate and one time with a TE buffer (10 mM Tris-HCl [pH8.0], 1 mM EDTA). The precipitated materials were eluted after incubation at 65 °C (10 minutes) in a buffer containing 50 mM Tris-HCl [pH7.5], 10 mM EDTA and 1% SDS. The decrosslinking step was performed overnight at 65 °C. We used the Invisorb Fragment CleanUp Kit to purify the DNA fragments.

### Quantitative PCR analysis (qPCR)

All the qPCR experiments realized for individual gene analysis were conducted with the BioRad CFX384 qPCR machine using the following parameters: Five minutes at 95°C followed by forty cycles (15 s at 95 °C + 15 s at 50 °C + 40 s at 72 °C), followed by ten minutes at 95°C. The oligonucleotides used for the qPCR reactions are listed in Table S2.

### Western blotting

Cells were grown in YPD and blocked in G2 as described previously. 10 ml cultures were collected and crosslinked with 1% formaldehyde for 10 minutes followed by quenching using 1.2M glycine. Pellets were resuspended in 5% TCA (5ml) and left on ice for 10 minutes, the pellets were snap frozen in liquid nitrogen and resuspended in 1 ml acetone at room temperature. The pellets are left to dry for at least 3 hours, and then resuspended in 100μl lysis buffer (50 mM Tris pH7.5, 1 mM EDTA pH7.5) 2.75 μl; 1M DTT, 20 μl 50x protease inhibitors). Glass beads were added to break cells in fast prep 3x45secs. 50 μl of 3xSDS sample buffer was added to the lysate followed by immediate heating at 95°C for 5 min, cooled and centrifuged before loading onto SDS-PAGE gels (8–10%). PAGE was carried out using a Bio-Rad Mini Trans-Blot System (Bio-Rad) in SDS running buffer (25 mM Tris, 190 mM glycine, 0.01% SDS). SDS-PAGE gels were transferred onto nitrocellulose membrane (0.45 μM, Amersham-GE Healthcare, Amersham, UK) in transfer buffer (25 mM Tris, 1.5% glycine, 0.02% SDS, 20% EtOH) in a Bio-Rad Mini Trans-Blot system. Membranes were blocked in 5% milk in PBS with 0.05% Tween20 (PBST) for at least 1 hr at room temperature before incubating in primary antibody in 2% milk/PBST overnight at 4°C. Membranes were washed in PBST three times for 15 min, incubated in secondary antibody in 2% milk/PBST for overnight at 4°C, and washed in PBST three times. Signals were detected with Amersham ECL detection reagents (RPN2105; Cytiva) and images were directly acquired with a ChemiDoc MP Imaging System (Bio-Rad). Primary antibodies used were rabbit anti-Smc3-K112,113Ac (a kind gift from Pr Adele Marston), mouse anti-V5 (Invitrogen), and rat anti-tubulin (Santa-Cruz). The ECL signals were quantified using Image Lab 6.0 (Bio-Rad). The quantification of the relative levels of proteins were calculated by normalizing the ratio signals of Smc3K112,113ac to tubulin. The statistical analyses were performed using one-tailed Mann-Whitney test.

### Data availability

The authors state that all data necessary for confirming the conclusions presented in the manuscript are represented fully within the manuscript.

## SUPPLEMENTAL MATERIAL

Click here for supplemental data file.

All supplemental data for this article are available online at www.cell-stress.com/researcharticles/2023a-ghaddar-cell-stress/.

## Abbreviations:

CAF-1: – chromatin assembly factor-1,
H3K56ac: – Histone H3 lysine 56 acetylation,
SCC: – sister chromatid cohesion,
UV: – ultraviolet,
CPT: – camptothecin,
HU: – hydroxyurea,
MMS: – methyl-methanesulfonate,
WT: – wild-type,
HR: – homologous recombination,
γH2AX: – phosphorylation of histone H2A on serine 129,
Polα: – DNA-polymerase α,
ts: – thermosensitive.
