# Supplementary material for: Chromatin assembly factor-1 preserves genome stability in ctf4Δ cells by promoting sister chromatid cohesion
Source: Cell Stress. 2023 Aug 14;7(9):69–89. doi: 10.15698/cst2023.09.289 (PMC10468696; doi:10.15698/cst2023.09.289)
Supplement: Supplementary file 1 [file ces-07-069-s01.pdf]

Supplemental Material

Chromatin assembly factor-1 preserves genome stability in *ctf4*Δ cells by promoting sister chromatid cohesion

Nagham Ghaddar[1], Pierre Luciano, Vincent Géli[1], and Yves Corda[1,*]
[1] Marseille Cancer Research Centre (CRCM), U1068 INSERM, UMR7258 CNRS, UM105 Aix Marseille Univ, Institut Paoli-Calmettes, Marseille, France. Ligue Nationale Contre le Cancer (Labeled Equip).
* Corresponding Author:
Yves Corda, Marseille Cancer Research Centre, U1068 INSERM, UMR7258 CNRS, UM105 Aix Marseille Univ, Institut Paoli-Calmettes, 27, Blvd Leï Roure, BP30059, 13273 Marseille Cedex 9, France; Phone: +33(0) 4 86 97 74 07; E-mail: yves.corda@inserm.fr

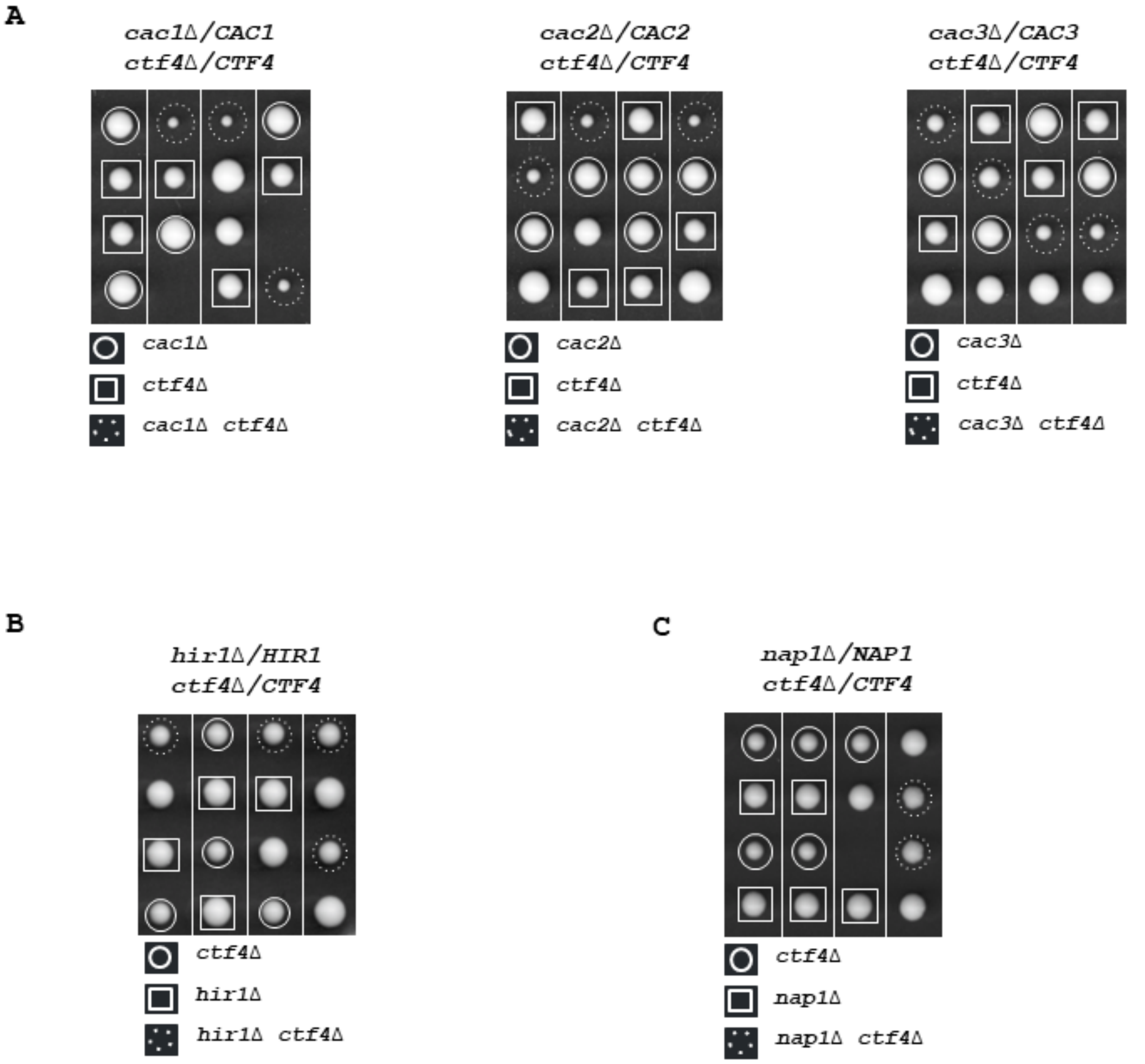

Figure S1, CAF-1 function is required for normal cell growth in absence of *CTF4*. (A) Tetrad dissection of the diploid strains *cac1Δ/CAC1 ctf4Δ/CTF4* (left), *cac2Δ/CAC2 ctf4Δ/CTF4* (center), and *cac3Δ/CAC3 ctf4Δ/CTF4* (right). In this and subsequent figures, the spores from a given tetrad are in vertical line on a YPD plate. One hundred tetrads were dissected. Four representative tetrads are shown after 3 days at 30°. *cac1Δ ctf4Δ*, *cac2Δ ctf4Δ*, and *cac3Δ ctf4Δ* mutants are indicated by dashed circles. (B) Tetrad dissection of the diploid strains *hir1Δ/HIR1 ctf4Δ/CTF4*. Fifty tetrads were dissected. (C) Tetrad dissection of the diploid strains *nap1Δ/NAP1 ctf4Δ/CTF4*. Fifty tetrads were dissected.

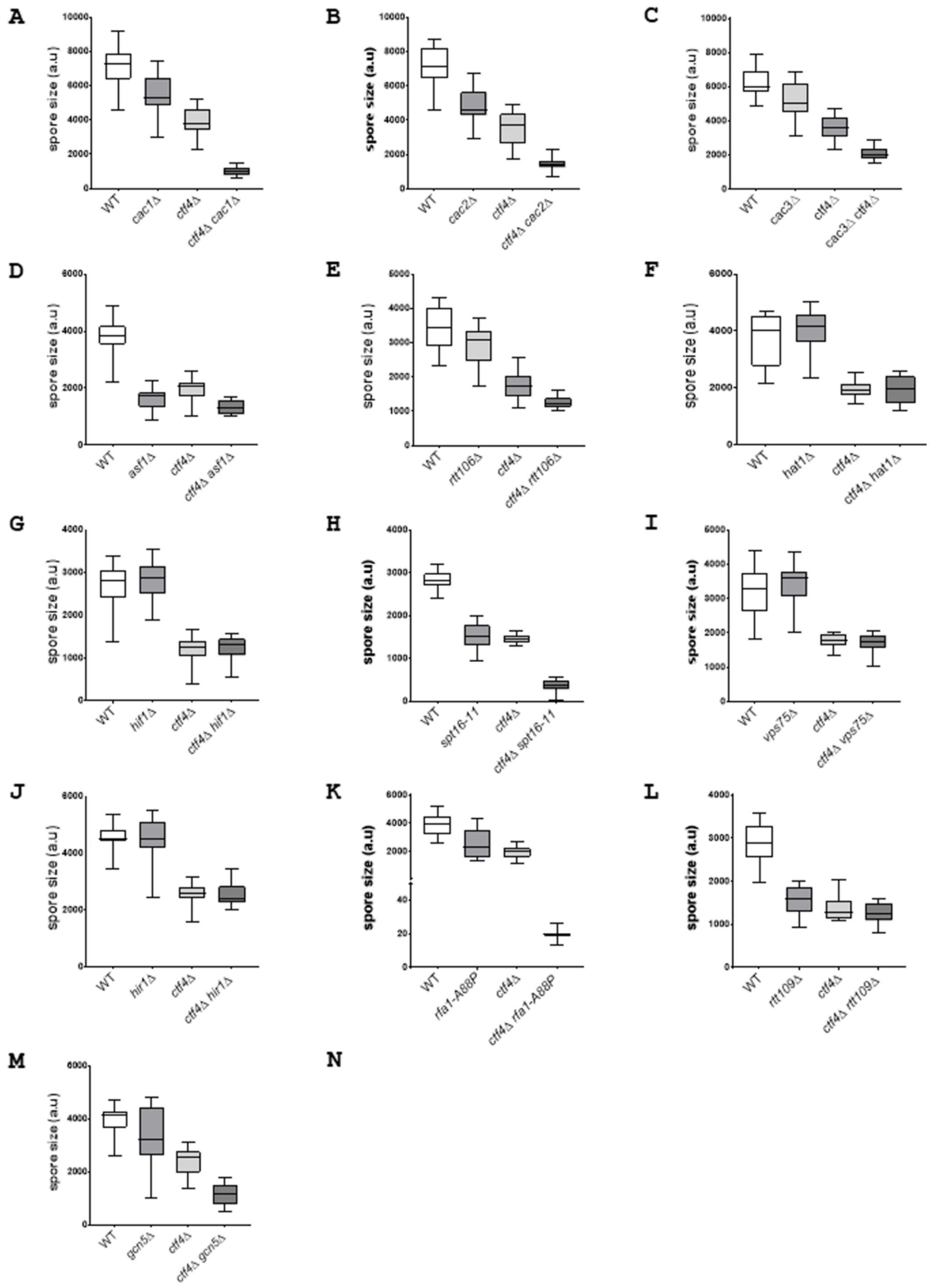

Figure S2, *ctf4Δ/CTF4* diploid strains heterozygous for the indicated mutations were sporulated, dissected, and the genotype of the viable spores was determined. The growth was quantified via image analysis (using ImageJ) after 3 days at 30° (25° for *spt16-11* mutant) on regular media (YPD). A minimum of 54 tetrades were analyzed for each of the diploids tested.

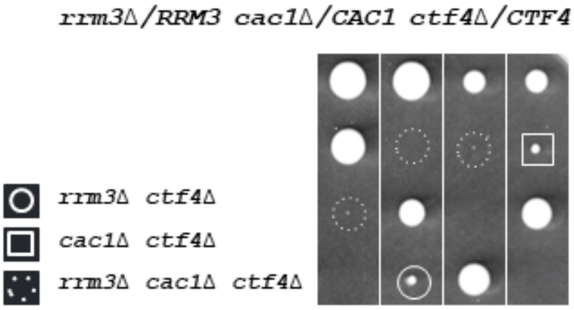

Figure S3, Rrm3 DNA helicase is required for the viability of yeast *cac1Δ ctf4Δ* cells. Tetrad dissection of the diploid strains *rrm3Δ/RRM3 cac1Δ/CAC1 ctf4Δ/CTF4*. Fifty tetrads were dissected. Four representative tetrads are shown after 3 days at 30° on YPD plate.

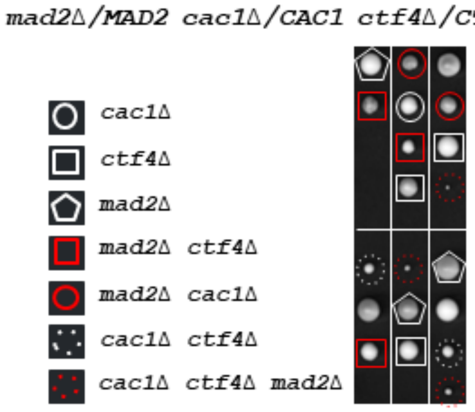

Figure S4, Genetic interaction of *MAD2*, with *CAC1* and *CTF4*. Fifty tetrads from the diploid strain *mad2Δ/MAD2 cac1Δ/CAC1 ctf4Δ/CTF4* were dissected and grown at 30° for 3 days on YPD plates.

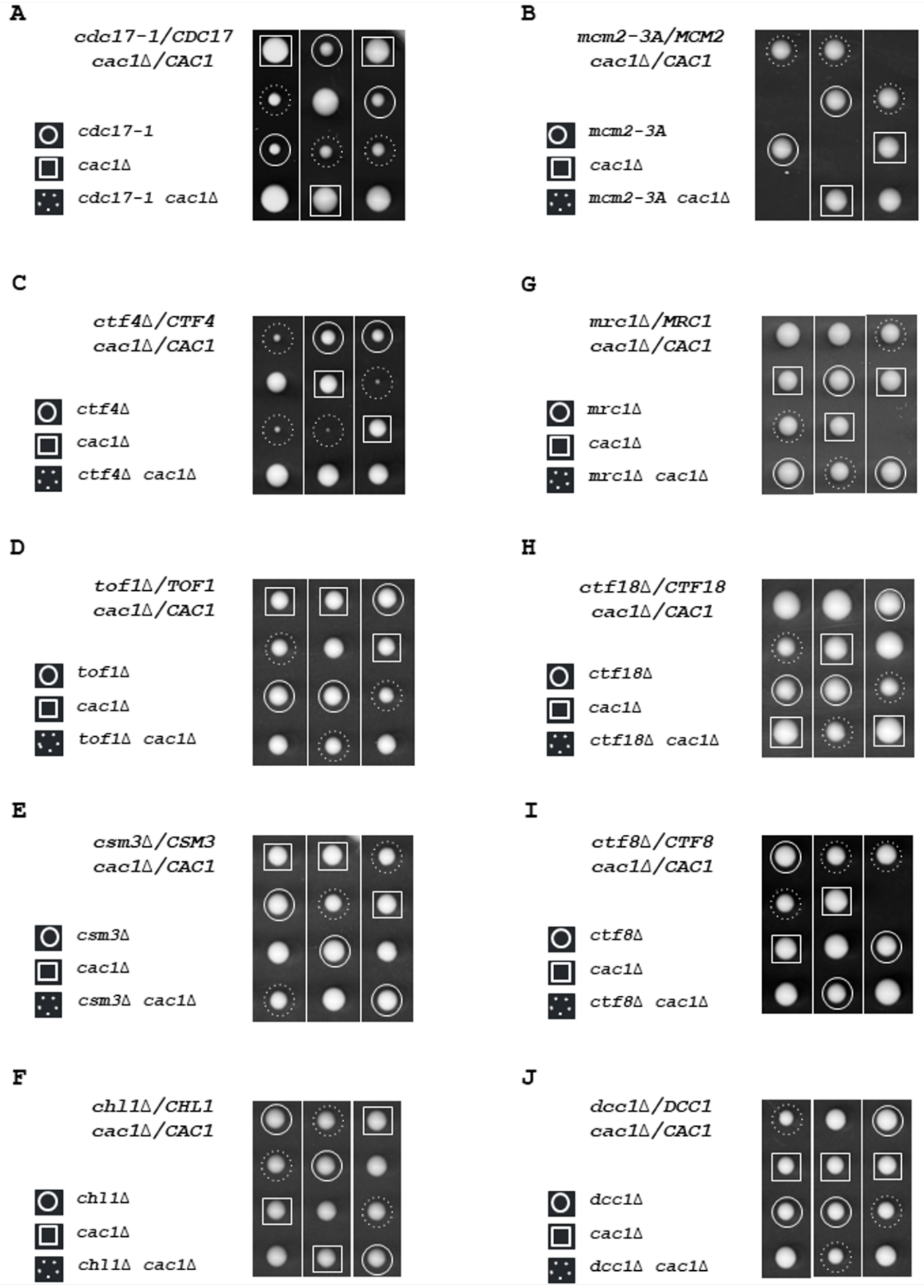

Figure S5, Assessment of the importance of different functions of Ctf4 in absence of *CAC1*. (A) Affecting the stability of the catalytic subunit of the DNA polymerase-alpha (Cdc17) does not affect the growth of *cac1Δ* mutant. The diploid strain *cdc17-1/CDC17 cac1Δ/CAC1* was sporulated and dissected. Dashed circles indicate the *cac1Δ cdc17-1* mutant. (B) Affecting the transfert of parental H3-H4 histones onto the lagging strand does not affect the growth of *cac1Δ* mutant. The diploid strain *mcm2-3A/MCM2 cac1Δ/CAC1* was sporulated and dissected. (C-J) Among the non-essential genes involved in the two parallel pathways for cohesion establishment at the replication fork, *CTF4* is the only gene required for the growth of *cac1Δ* mutant. The diploid strains *ctf4Δ/CTF4 cac1Δ/CAC1* (C), *tof1Δ/TOF1 cac1Δ/CAC1 ctf4Δ/CTF4* (D), *csm3Δ/CSM3 cac1Δ/CAC1 ctf4Δ/CTF4* (E), *chl1Δ/CHL1 cac1Δ/CAC1 ctf4Δ/CTF4* (F), *mrc1Δ/MRC1 cac1Δ/CAC1* (G), *ctf18Δ/CTF18 cac1Δ/CAC1* (H), *ctf8Δ/CTF8 cac1Δ/CAC1* (I), and *dcc1Δ/DCC1 cac1Δ/CAC1* (J), were sporulated and dissected. Tetrads were displayed vertically on a YPD plate and incubated at 30°. Fifty tetrads were dissected for each of the diploids. Three representative tetrads are shown for each dissection.

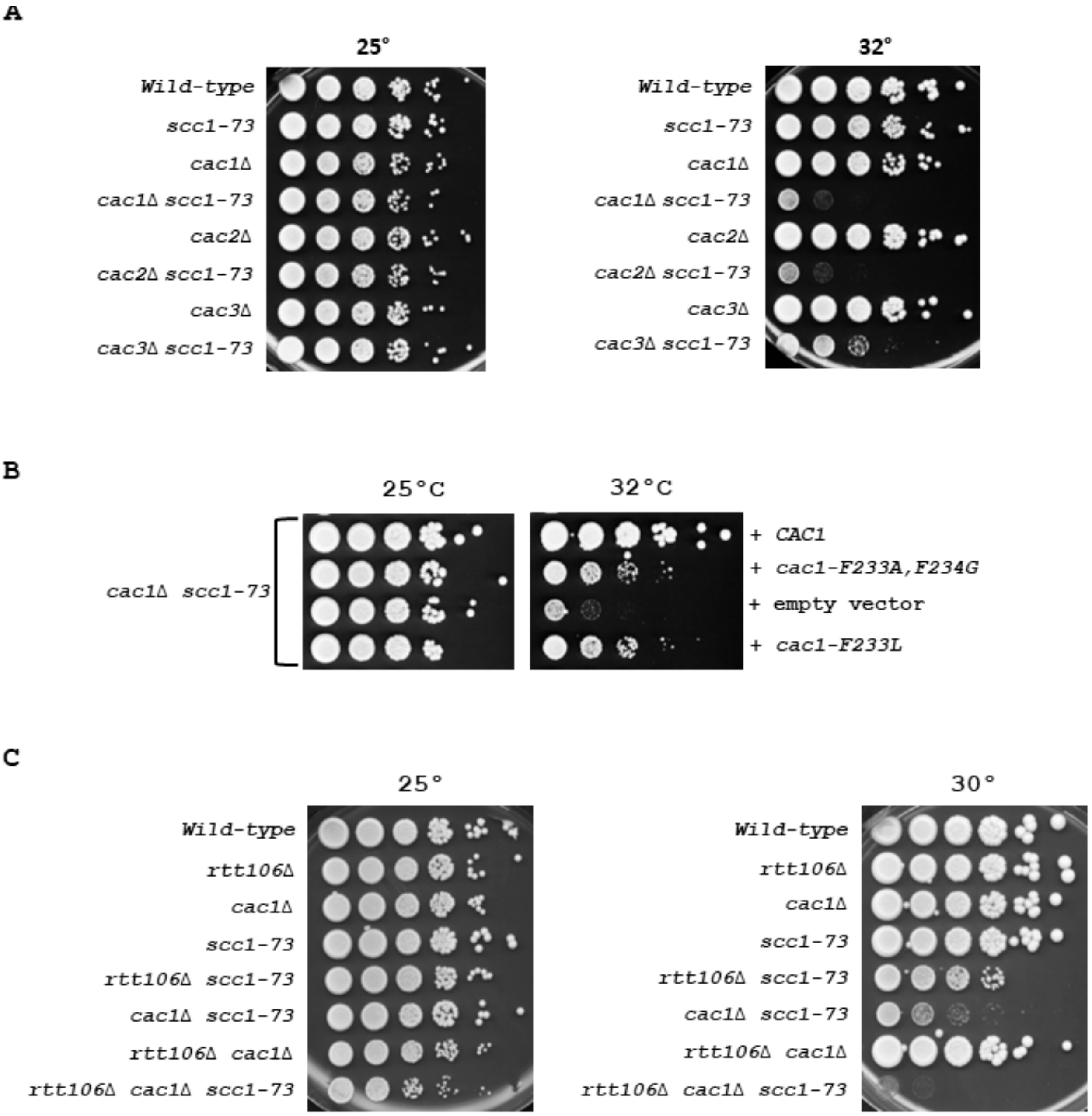

Figure S6 Proper replication coupled nucleosome assembly is essential for cell viability in presence of cohesin defects. (A) Each of CAF-1 subunits is required for cell viability in presence of cohesin defects. Tenfold serial dilutions of exponentially growing cells with the indicated mutations were spotted onto YPD plates and incubated at 25°, or 32° for 3 days. (B) CAF-1 recruitment to replication fork is required for growth in cells affected in cohesin function. *cac1Δ scc1-73* cells expressing from a centromeric plasmid either wild-type *CAC1*, *cac1-F233A,F234G* mutant, or *cac1-F233L* mutant both affected in Cac1 interaction with PCNA and consequently deficient in CAF-1 DNA-replication-linked nucleosome assembly function, were analyzed for growth after 3 days incubation at 25°, or 32°. (C) CAF-1 and Rtt106 chromatin assembly pathways are both required for normal growth in presence of cohesin defects. Genetic interactions among *SCC1*, *RTT106*, and *CAC1* were assessed after 3 days incubation at 25°, or 30°, by spotting a ten fold series dilution of cells of the indicated genotype onto YPD.

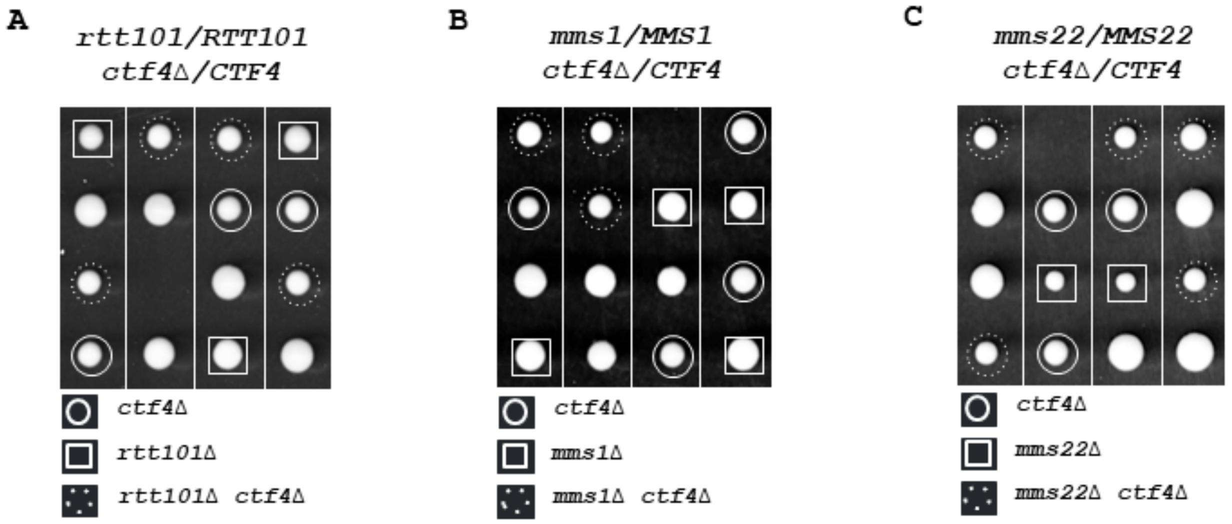

Figure S7, The Rtt101-Mms1-Mms22 E3 ubiquitin ligase complex is not required for the growth of *ctf4Δ* cells. Tetrads from diploids heterozygous for *rtt101Δ/RTT101 ctf4Δ/CTF4* (A), for *mms1Δ/MMS1 ctf4Δ/CTF4* (B), and for *mms22Δ/MMS22 ctf4Δ/CTF4* (C), were dissected and analyzed after 3 days at 30°. For each diploids, fifty tetrads were dissected. Four representative tetrads are shown for each dissection.

Table S1: Strains used in this study (All the strains were based on W303)

YVC600	*MATa/MATα ade2-1/ade2-1 can1-100/can1-100 his3-11,15/his3-11,15 leu2-3,112/leu2-3,112 trp1-1/trp1-1 ura3-1/ura3-1 cac1Δ(::HphMX)/CAC1 rtt106Δ(::LEU2)/RTT106 ctf4Δ(::TRP1)/CTF4*
YVC601	*MATa/MATα ade2-1/ade2-1 can1-100/can1-100 his3-11,15/his3-11,15 leu2-3,112/leu2-3,112 trp1-1/trp1-1 ura3-1/ura3-1 ctf4Δ(::URA3)/CTF4 hht1-hhf1Δ(::LEU2) hht2-hhf2Δ(::KanMX3) hht1(K9,14,18,23,27R)-HHF1*/pRS414(*TRP1*)
YVC602	*MATa/MATα ade2-1/ade2-1 can1-100/can1-100 his3-11,15/his3-11,15 leu2-3,112/leu2-3,112 trp1-1/trp1-1 ura3-1/ura3-1 ctf4Δ(::TRP1)/CTF4 hht1-hhf1Δ(::LEU2) hht2-hhf2Δ(::KanMX3) hhf2(K5,8,12R)-HHT2*/pRS313(*HIS3*)
YVC603	*MATa/MATα ade2-1/ade2-1 can1-100/can1-100 his3-11,15/his3-11,15 leu2-3,112/leu2-3,112 trp1-1/trp1-1 ura3-1/ura3-1 cac1Δ(::HphMX)/CAC1 ctf4Δ(::TRP1)/CTF4*
YVC604	*MATa/MATα ade2-1/ade2-1 can1-100/can1-100 his3-11,15/his3-11,15 leu2-3,112/leu2-3,112 trp1-1/trp1-1 ura3-1/ura3-1 cac2Δ(::TRP1/CAC2 ctf4Δ(::URA3)/CTF4*
YVC605	*MATa/MATα ade2-1/ade2-1 can1-100/can1-100 his3-11,15/his3-11,15 leu2-3,112/leu2-3,112 trp1-1/trp1-1 ura3-1/ura3-1 cac3Δ(::TRP1/CAC3 ctf4Δ(::URA3)/CTF4*
YVC606	*MATa/MATα ade2-1/ade2-1 can1-100/can1-100 his3-11,15/his3-11,15 leu2-3,112/leu2-3,112 trp1-1/trp1-1 ura3-1/ura3-1 hir1Δ(::TRP1/HIR1 ctf4Δ(::URA3)/CTF4*
YVC607	*MATa/MATα ade2-1/ade2-1 can1-100/can1-100 his3-11,15/his3-11,15 leu2-3,112/leu2-3,112 trp1-1/trp1-1 ura3-1/ura3-1 nap1Δ(::TRP1/NAP1 ctf4Δ(::URA3)/CTF4*
YVC608	*MATa/MATα ade2-1/ade2-1 can1-100/can1-100 his3-11,15/his3-11,15 leu2-3,112/leu2-3,112 trp1-1/trp1-1 ura3-1/ura3-1 asf1Δ(::KanMX6)/ASF1 ctf4Δ(::TRP1)/CTF4*
YVC609	*MATa/MATα ade2-1/ade2-1 can1-100/can1-100 his3-11,15/his3-11,15 leu2-3,112/leu2-3,112 trp1-1/trp1-1 ura3-1/ura3-1 hat1Δ(::TRP1/HAT1 ctf4Δ(::URA3)/CTF4*
YVC610	*MATa/MATα ade2-1/ade2-1 can1-100/can1-100 his3-11,15/his3-11,15 leu2-3,112/leu2-3,112 trp1-1/trp1-1 ura3-1/ura3-1 hif1Δ(::TRP1)/HIF1 ctf4Δ(::URA3)/CTF4*
YVC611	*MATa/MATα ade2-1/ade2-1 can1-100/can1-100 his3-11,15/his3-11,15 leu2-3,112/leu2-3,112 trp1-1/trp1-1 ura3-1/ura3-1 spt16-1/SPT16 ctf4Δ(::TRP1)/CTF4*

YVC612	*MATa/MATα ade2-1/ade2-1 can1-100/can1-100 his3-11,15/his3-11,15 leu2-3,112/leu2-3,112 trp1-1/trp1-1 ura3-1/ura3-1 vps75Δ(::TRP1)/VPS75 ctf4Δ(::URA3)/CTF4*
YVC613	*MATa/MATα ade2-1/ade2-1 can1-100/can1-100 his3-11,15/his3-11,15 leu2-3,112/leu2-3,112 trp1-1/trp1-1 ura3-1/ura3-1 rfa1-A88P/RFA1 ctf4Δ(::TRP1)/CTF4*
YVC614	*MATa/MATα ade2-1/ade2-1 can1-100/can1-100 his3-11,15/his3-11,15 leu2-3,112/leu2-3,112 trp1-1/trp1-1 ura3-1/ura3-1 rtt109Δ(::LEU2)/RTT109 ctf4Δ(::URA3)/CTF4*
YVC615	*MATa/MATα ade2-1/ade2-1 can1-100/can1-100 his3-11,15/his3-11,15 leu2-3,112/leu2-3,112 trp1-1/trp1-1 ura3-1/ura3-1 gcn5Δ(::TRP1)/GCN5 ctf4Δ(::URA3)/CTF4*
YVC616	*MATa/MATα ade2-1/ade2-1 can1-100/can1-100 his3-11,15/his3-11,15 leu2-3,112/leu2-3,112 trp1-1/trp1-1 ura3-1/ura3-1 elg3Δ(::TRP1)/ELG3 ctf4Δ(::URA3)/CTF4*
YVC617	*ade2-1 can1-100 his3-11,15 leu2-3,112 trp1-1 ura3-1 cac1Δ(::HphMX)*
YVC618	*ade2-1 can1-100 his3-11,15 leu2-3,112 trp1-1 ura3-1 ctf4Δ(::TRP1)*
YVC619	*ade2-1 can1-100 his3-11,15 leu2-3,112 trp1-1 ura3-1 cac1Δ(::HphMX) ctf4Δ(::TRP1)*
YVC620	*MATa/MATα ade2-1/ade2-1 can1-100/can1-100 his3-11,15/his3-11,15 leu2-3,112/leu2-3,112 trp1-1/trp1-1 ura3-1/ura3-1 rrm3Δ(::NatMX)/RRM3 cac1Δ(::HphMX)/CAC1 ctf4Δ(::TRP1)/CTF4*
YVC621	*MATa/MATα ade2-1/ade2-1 can1-100/can1-100 his3-11,15/his3-11,15 leu2-3,112/leu2-3,112 trp1-1/trp1-1 ura3-1/ura3-1 pif1Δ(::KanMX6)/PIF1 cac1Δ(::HphMX)/CAC1 ctf4Δ(::TRP1)/CTF4*
YVC622	*MATa/MATα ade2-1/ade2-1 can1-100/can1-100 his3-11,15/his3-11,15 leu2-3,112/leu2-3,112 trp1-1/trp1-1 ura3-1/ura3-1 mec1Δ(:: TRP1)/MEC1 sml1Δ(::HIS3)/SML1 cac1Δ(::HphMX)/CAC1 ctf4Δ(::URA)/CTF4*
YVC623	*MATa/MATα ade2-1/ade2-1 can1-100/can1-100 his3-11,15/his3-11,15 leu2-3,112/leu2-3,112 trp1-1/trp1-1 ura3-1/ura3-1 rad53-K227A(::KanMX6)/RAD53 cac1Δ(::HphMX)/CAC1 ctf4Δ(::TRP1)/CTF4*
YVC624	*can1-100 his3-11,15 leu2-3,112 trp1-1 ura3-1 rad52-YFP*
YVC625	*can1-100 his3-11,15 leu2-3,112 trp1-1 ura3-1 rad52-YFP cac1Δ(::HphMX)*
YVC626	*can1-100 his3-11,15 leu2-3,112 trp1-1 ura3-1 rad52-YFP ctf4Δ(::TRP1)*
YVC627	*can1-100 his3-11,15 leu2-3,112 trp1-1 ura3-1 rad52-YFP cac1Δ(::HphMX) ctf4Δ(::TRP1)*
YVC628	*can1-100 his3-11,15 leu2-3,112 trp1-1 ura3-1 rfa1-CFP*
YVC629	*can1-100 his3-11,15 leu2-3,112 trp1-1 ura3-1 rfa1-CFP cac1Δ(::HphMX)*
YVC630	*can1-100 his3-11,15 leu2-3,112 trp1-1 ura3-1 rfa1-CFP ctf4Δ(::TRP1)*
YVC631	*can1-100 his3-11,15 leu2-3,112 trp1-1 ura3-1 rfa1-CFP cac1Δ(::HphMX) ctf4Δ(::TRP1)*

YVC632	*MATa / MATα ade2-1/ade2-1 can1-100/can1-100 his3-11,15/his3-11,15 leu2-3,112/leu2-3,112 trp1-1/trp1-1 ura3-1/ura3-1 mad2Δ(:: TRP1)/MAD2 cac1Δ(::HphMX)/CAC1 ctf4Δ(::URA)/CTF4*
YVC633	*MATa / MATα ade2-1/ade2-1 can1-100/can1-100 his3-11,15/his3-11,15 leu2-3,112/leu2-3,112 trp1-1/trp1-1 ura3-1/ura3-1 cdc17-1/CDC17 cac1Δ(::HphMX)/CAC1*
YVC634	*MATa / MATα ade2-1/ade2-1 can1-100/can1-100 his3-11,15/his3-11,15 leu2-3,112/leu2-3,112 trp1-1/trp1-1 ura3-1/ura3-1 mcm2-3A/MCM2 cac1Δ(::HphMX)/CAC1*
YVC635	*MATa / MATα ade2-1/ade2-1 can1-100/can1-100 his3-11,15/his3-11,15 leu2-3,112/leu2-3,112 trp1-1/trp1-1 ura3-1/ura3-1 tof1Δ(:: TRP1)/TOF1 cac1Δ(::HphMX)/CAC1 ctf4Δ(::URA3)/CTF4*
YVC636	*MATa / MATα ade2-1/ade2-1 can1-100/can1-100 his3-11,15/his3-11,15 leu2-3,112/leu2-3,112 trp1-1/trp1-1 ura3-1/ura3-1 csm3Δ(:: URA)/CSM3 cac1Δ(::HphMX)/CAC1 ctf4Δ(::URA3)/CTF4*
YVC637	*MATa / MATα ade2-1/ade2-1 can1-100/can1-100 his3-11,15/his3-11,15 leu2-3,112/leu2-3,112 trp1-1/trp1-1 ura3-1/ura3-1 chl1Δ(:: URA)/CHL1 cac1Δ(::HphMX)/CAC1 ctf4Δ(::URA3)/CTF4*
YVC638	*MATa / MATα ade2-1/ade2-1 can1-100/can1-100 his3-11,15/his3-11,15 leu2-3,112/leu2-3,112 trp1-1/trp1-1 ura3-1/ura3-1 mrc1Δ(::NatMX)/MRC1 cac1Δ(::HphMX)/CAC1*
YVC639	*MATa / MATα ade2-1/ade2-1 can1-100/can1-100 his3-11,15/his3-11,15 leu2-3,112/leu2-3,112 trp1-1/trp1-1 ura3-1/ura3-1 ctf18Δ(:: TRP1)/CTF18 cac1Δ(::HphMX)/CAC1*
YVC640	*MATa / MATα ade2-1/ade2-1 can1-100/can1-100 his3-11,15/his3-11,15 leu2-3,112/leu2-3,112 trp1-1/trp1-1 ura3-1/ura3-1 ctf8Δ(:: TRP1)/CTF8 cac1Δ(::HphMX)/CAC1*
YVC641	*MATa / MATα ade2-1/ade2-1 can1-100/can1-100 his3-11,15/his3-11,15 leu2-3,112/leu2-3,112 trp1-1/trp1-1 ura3-1/ura3-1 dcc1Δ(:: TRP1)/DCC1 cac1Δ(::HphMX)/CAC1*
YVC642	*MATa / MATα ade2-1/ade2-1 can1-100/can1-100 his3-11,15/his3-11,15 leu2-3,112/leu2-3,112 trp1-1/trp1-1 ura3-1/ura3-1 eco1Δ(:: URA3)/ECO1 rad61Δ(:: TRP1)/RAD61 cac1Δ(::KanMX6)/CAC1*
YVC643	*ade2-1 can1-100 his3-11,15 leu2-3,112 trp1-1 ura3-1 rad61Δ(::TRP1)*
YVC644	*ade2-1 can1-100 his3-11,15 leu2-3,112 trp1-1 ura3-1 rad61Δ(::TRP1) cac1Δ(::KanMX6)*
YVC645	*ade2-1 can1-100 his3-11,15 leu2-3,112 trp1-1 ura3-1 eco1Δ(::URA) rad61Δ(::TRP1)*
YVC646	*ade2-1 can1-100 his3-11,15 leu2-3,112 trp1-1 ura3-1 eco1Δ(::URA) rad61Δ(::TRP1) cac1Δ(::KanMX6)*
YVC647	*ade2-1 can1-100 his3-11,15 leu2-3,112 trp1-1 ura3-1 cac1Δ(::KanMX6)*
YVC648	*ade2-1 can1-100 his3-11,15 leu2-3,112 trp1-1 ura3-1 eco1-1*
YVC649	*ade2-1 can1-100 his3-11,15 leu2-3,112 trp1-1 ura3-1 eco1-1 cac1Δ(::KanMX6)*
YVC650	*ade2-1 can1-100 his3-11,15 leu2-3,112 trp1-1 ura3-1 scc2-4*

YVC651	*ade2-1 can1-100 his3-11,15 leu2-3,112 trp1-1 ura3-1 scc2-4 cac1Δ(::HphMX)*
YVC652	*ade2-1 can1-100 his3-11,15 leu2-3,112 trp1-1 ura3-1 scc1-73*
YVC653	*ade2-1 can1-100 his3-11,15 leu2-3,112 trp1-1 ura3-1 scc1-73 cac1Δ(::HphMX)*
YVC654	*ade2-1 can1-100 his3-11,15 leu2-3,112 trp1-1 ura3-1 cac2Δ(::TRP1)*
YVC655	*ade2-1 can1-100 his3-11,15 leu2-3,112 trp1-1 ura3-1 scc1-73 cac2Δ(::TRP1)*
YVC656	*ade2-1 can1-100 his3-11,15 leu2-3,112 trp1-1 ura3-1 cac3Δ(::TRP1)*
YVC657	*ade2-1 can1-100 his3-11,15 leu2-3,112 trp1-1 ura3-1 scc1-73 cac3Δ(::TRP1)*
YVC658	*ade2-1 can1-100 his3-11,15 leu2-3,112 trp1-1 ura3-1 rtt106Δ(::LEU2)*
YVC659	*ade2-1 can1-100 his3-11,15 leu2-3,112 trp1-1 ura3-1 scc1-73 rtt106Δ(::LEU2)*
YVC660	*ade2-1 can1-100 his3-11,15 leu2-3,112 trp1-1 ura3-1 rtt106Δ(::LEU2) cac1Δ(::HphMX)*
YVC661	*ade2-1 can1-100 his3-11,15 leu2-3,112 trp1-1 ura3-1 scc1-73 rtt106Δ(::LEU2) cac1Δ(::HphMX)*
YVC662	*ade2-1 can1-100 his3-11,15 leu2-3,112 trp1-1 ura3-1 rtt101Δ(::URA3)*
YVC663	*ade2-1 can1-100 his3-11,15 leu2-3,112 trp1-1 ura3-1 rtt101Δ(::URA3) cac1Δ(::HphMX)*
YVC664	*ade2-1 can1-100 his3-11,15 leu2-3,112 trp1-1 ura3-1 mms1Δ(::TRP1)*
YVC665	*ade2-1 can1-100 his3-11,15 leu2-3,112 trp1-1 ura3-1 mms1Δ(::TRP1) cac1Δ(::HphMX)*
YVC666	*ade2-1 can1-100 his3-11,15 leu2-3,112 trp1-1 ura3-1 mms22Δ(::TRP1)*
YVC667	*ade2-1 can1-100 his3-11,15 leu2-3,112 trp1-1 ura3-1 mms22Δ(::TRP1) cac1Δ(::HphMX)*
YVC668	*ade2-1 can1-100 his3-11,15 leu2-3,112 trp1-1 ura3-1 rtt101Δ(::URA3) scc1-73*
YVC669	*ade2-1 can1-100 his3-11,15 leu2-3,112 trp1-1 ura3-1 mms1Δ(::TRP1) scc1-73*
YVC670	*ade2-1 can1-100 his3-11,15 leu2-3,112 trp1-1 ura3-1 mms22Δ(::TRP1) scc1-73*
YVC671	*ade2-1 can1-100 his3-11,15 leu2-3,112 trp1-1 ura3-1 rtt101Δ(::URA3) cac1Δ(::HphMX) scc1-73*
YVC672	*ade2-1 can1-100 his3-11,15 leu2-3,112 trp1-1 ura3-1 mms1Δ(::TRP1) cac1Δ(::HphMX) scc1-73*
YVC673	*ade2-1 can1-100 his3-11,15 leu2-3,112 trp1-1 ura3-1 mms22Δ(::TRP1) cac1Δ(::HphMX) scc1-73*
YVC229	*MAT*a*/MAT*α *ade2-1/ade2-1 can1-100/can1-100 his3-11,15/his3-11,15 leu2-3,112/leu2-3,112 trp1-1/trp1-1 ura3-1/ura3-1 rrm3Δ(::NatMX)/RRM3 rtt101Δ(::URA3)/RTT101 ctf4Δ(::TRP1)/CTF4*
YVC230	*MAT*a*/MAT*α *ade2-1/ade2-1 can1-100/can1-100 his3-11,15/his3-11,15 leu2-3,112/leu2-3,112 trp1-1/trp1-1 ura3-1/ura3-1 rrm3Δ(::NatMX)/RRM3 mms1Δ(::TRP1)/MMS1 ctf4Δ(::TRP1)/CTF4*
YVC231	*MAT*a*/MAT*α *ade2-1/ade2-1 can1-100/can1-100 his3-11,15/his3-11,15 leu2-3,112/leu2-3,112 trp1-1/trp1-1 ura3-1/ura3-1 rrm3Δ(::NatMX)/RRM3 mms22Δ(::TRP1)/MMS22 ctf4Δ(::TRP1)/CTF4*
SBY885	*MAT*a *ade2-1 can1-100 bar1-1 leu2-3,112 lys2Δ trp1-1 ura3-1 his3-11 :pCUP1GFP12-LacI12 ::HIS cenIII-Lac0128 :TRP1 (S. Biggins)*

YVC680	*MATa ade2-1 can1-100 bar1-1 leu2-3,112 lys2Δ trp1-1 ura3-1 his3-11 :pCUP1GFP12-LacI12 ::HIS cenIII-Lac0128 :TRP1) cac1Δ(::HphMX)*
YVC681	*MATa ade2-1 can1-100 bar1-1 leu2-3,112 lys2Δ trp1-1 ura3-1 his3-11 :pCUP1GFP12-LacI12 ::HIS cenIII-Lac0128 :TRP1) ctf4Δ(::URA3)*
YVC682	*MATa ade2-1 can1-100 bar1-1 leu2-3,112 lys2Δ trp1-1 ura3-1 his3-11 :pCUP1GFP12-LacI12 ::HIS cenIII-Lac0128 :TRP1) cac1Δ(::HphMX) ctf4Δ(::URA3)*
PP1418	*MATa, ade2–1, trp1–1, can1–100, leu2–3,112, his3–11,15, ura3, GAL, psi+, RAD5, Scc1–PK9–TRP, URA3::GPD–TK7*
PP1628	*MATa, ade2–1, trp1–1, can1–100, leu2–3,112, his3–11,15, ura3, GAL, RAD5+, Scc2–PK9–TRP, URA3::GPD–TK7* (Delamarre et al. 2020)
YVC683	*ade2-1 can1-100 his3-11,15 leu2-3,112 trp1-1 ura3-1 SCC1-Pk9 (::TRP1)*
YVC684	*ade2-1 can1-100 his3-11,15 leu2-3,112 trp1-1 ura3-1 SCC1-Pk9 (::TRP1) cac1Δ(::HphMX)*
YVC685	*ade2-1 can1-100 his3-11,15 leu2-3,112 trp1-1 ura3-1 SCC1-Pk9 (::TRP1) chk1Δ(::KanMX6)*
YVC686	*ade2-1 can1-100 his3-11,15 leu2-3,112 trp1-1 ura3-1 SCC1-Pk9 (::TRP1) chk1Δ(::KanMX6) cac1Δ(::HphMX)*
YVC687	*ade2-1 can1-100 his3-11,15 leu2-3,112 trp1-1 ura3-1 SCC1-Pk9 (::TRP1) scc2-4*
YVC688	*ade2-1 can1-100 his3-11,15 leu2-3,112 trp1-1 ura3-1 SCC1-Pk9 (::TRP1) scc2-4 cac1Δ(::HphMX)*
YVC689	*ade2-1 can1-100 his3-11,15 leu2-3,112 trp1-1 ura3-1 SCC1-Pk9 (::TRP1) ctf4Δ(::URA3)*
YVC690	*ade2-1 can1-100 his3-11,15 leu2-3,112 trp1-1 ura3-1 SCC1-Pk9 (::TRP1) ctf4Δ(::URA3) cac1Δ(::HphMX)*
YVC1000	*ade2-1 can1-100 his3-11,15 leu2-3,112 trp1-1 ura3-1 21xTer(::URA3)*
YVC1001	*ade2-1 can1-100 his3-11,15 leu2-3,112 trp1-1 ura3-1 cac1Δ(::HphMX) 21xTer(::URA3)*
YVC1002	*ade2-1 can1-100 his3-11,15 leu2-3,112 trp1-1 ura3-1 ctf4Δ(::KanMX6) 21xTer(::URA3)*
YVC1003	*ade2-1 can1-100 his3-11,15 leu2-3,112 trp1-1 ura3-1 21xTer(::URA3) cac1Δ(::HphMX) ctf4Δ(::KanMX6) 21xTer(::URA3)*

Table S2: Primers used in this study

Primers	Sequence (5' -3')
POA1-F	AAACGGCCACATCAAATACC
POA1-R	TCCAAGGGACTCCGAATATG
MRP10-F	ACCCCCTCTTCCCAGACTAA
MRP10-R	CCAGCACATTTAGGGCTCAT
MET10-F	ACTTGTGTGGCCCTACTTGG
MET10-R	CGACTTTGATGCCTCTTTCC
CEN3-F	CGCCACTTTAACAAATGTGC
CEN3-R	GCAGAACCACCGTAGCAGTT
CEN9-F	TGTCACCTGGCTGTTTTGAG
CEN9-R	TGGGTAATGTCAGCTGTGGA
GLT1-F	TTTGACCCCAGCACATGTTA
GLT1-F	GGGTGTGGAGTTTGTGGTCT
RPL23B-F	CCGTCAAGCTAAGTCTTGGAGAAG
RPL23B-R	CCTTAGGATTAGCGATGACACCAG
RPL34A-F	GTCTTGTGGGTCTTGGAAACAG
RPL34A-R	CAAAGTGTGGTGACTGTGGTAG
PUG1-F	GCGGCAAGCTCATCCTAAAT
PUG1-R	CCCACAATTGATTCGGTAGG
tH(GUG)E1-F	GAAACCCTGGTTCGATTCTAGGAG
tH(GUG)E1-R	GCTCTCATGATCACCACATCTGAC
CIN8-F	AGGGCACAACTAGATAAACAGCA
CIN8-R	GGGCCATTTGCATTACCTCAGTCA

Table S3: Plasmids used in this study

pRS314-*CAC1* (Paul D. Kaufman)

pRS314-*cac1-F233L* (Paul D. Kaufman)

pRS314-*cac1-F233A,F234G* (Paul D. Kaufman)

pG413-*HA-TUS*
